# Interrupting peptidoglycan deacetylation during *Bdellovibrio* predator-prey interaction prevents ultimate destruction of prey wall, liberating bacterial-ghosts

**DOI:** 10.1038/srep26010

**Published:** 2016-05-23

**Authors:** Carey Lambert, Thomas R. Lerner, Nhat Khai Bui, Hannah Somers, Shin-Ichi Aizawa, Susan Liddell, Ana Clark, Waldemar Vollmer, Andrew L. Lovering, R. Elizabeth Sockett

**Affiliations:** 1 Centre for Genetics and Genomics, School of Life Sciences, University of Nottingham, Medical School, Queen’s Medical Centre, Nottingham, NG7 2UH, UK; 2 The Francis Crick Institute, Mill Hill Laboratory, The Ridgeway, Mill Hill, London, NW7 1AA, UK; 3 The Centre for Bacterial Cell Biology, Baddiley Clark Building, Medical School, Newcastle University, Richardson Road, Newcastle upon Tyne, NE2 4AX, UK; 4 Department of Life Sciences, Prefectural University of Hiroshima, Shobara, Hiroshima, 727-0023, Japan; 5 School of Biosciences, University of Nottingham, Sutton Bonington, Leicestershire, LE12 5RD, UK; 6 Institute for Microbiology and Infection, School of Biosciences, University of Birmingham, Birmingham, B15 2TT, UK

## Abstract

The peptidoglycan wall, located in the periplasm between the inner and outer membranes of the cell envelope in Gram-negative bacteria, maintains cell shape and endows osmotic robustness. Predatory *Bdellovibrio* bacteria invade the periplasm of other bacterial prey cells, usually crossing the peptidoglycan layer, forming transient structures called bdelloplasts within which the predators replicate. Prey peptidoglycan remains intact for several hours, but is modified and then degraded by escaping predators. Here we show predation is altered by deleting two *Bdellovibrio* N-acetylglucosamine (GlcNAc) deacetylases, one of which we show to have a unique two domain structure with a novel regulatory”plug”. Deleting the deacetylases limits peptidoglycan degradation and rounded prey cell “ghosts” persist after mutant-predator exit. Mutant predators can replicate unusually in the periplasmic region between the peptidoglycan wall and the outer membrane rather than between wall and inner-membrane, yet still obtain nutrients from the prey cytoplasm. Deleting two further genes encoding DacB/PBP4 family proteins, known to decrosslink and round prey peptidoglycan, results in a quadruple mutant *Bdellovibrio* which leaves prey-shaped ghosts upon predation. The resultant bacterial ghosts contain cytoplasmic membrane within bacteria-shaped peptidoglycan surrounded by outer membrane material which could have promise as “bacterial skeletons” for housing artificial chromosomes.

*Bdellovibrio bacteriovorus* is a small predatory bacterium which enters the periplasm of Gram-negative prey cells forming a transient structure called a bdelloplast. The rounded prey-bdelloplast is dead, but osmotically stable while the *Bdellovibrio* replicate within it, consuming its cellular contents and finally bursting/exiting post-replication. This re-sculpting of the prey cell and its peptidoglycan wall requires precise spatial and temporal control by novel modifying enzymes. Previously we discovered^1^,^2^ that two predatory enzymes (Bd3459 and Bd0816) of the DacB/Penicillin-binding-protein (PBP) 4 family cause rounding of the Gram-negative prey cell walls (also called sacculi) and that this allowed rapid invasion of the prey-bacterial periplasm and establishment of an infected bacterial prey-bdelloplast with a growing *Bdellovibrio* inside. It had been hypothesised in the 1970s that deacetylation of prey peptidoglycan sacculi by *B. bacteriovorus* would render them lysozyme and lytic transglycosylase-resistant so that they were stable during predatory digestion of contents and replication^3^,^4^. Hence, deacetylation could act as a chemical feature demarking prey wall material in the predator/prey system to keep it stable. Peptidoglycan *N*-deacetylase activity was detected biochemically by those workers^3^,^4^ in extracts from crushed bdelloplasts, but no specific enzymes were discovered. We identified two related genes (*bd0468* and *bd3279*) in the *B. bacteriovorus* HD100 genome whose products had homology to one domain of the peptidoglycan deacetylase PgdA of *Streptococcus pneumoniae.* PgdA modifies self-wall to protect *S. pneumoniae* against human lysozyme action in the nasopharynx^5^.

Here we show that both genes are expressed early in predation and act upon the GlcNAc residues of the prey cell to modify the prey peptidoglycan structure. In contrast to early ideas of solely stabilising prey peptidoglycan; the modification caused by the Bd0468 and Bd3279 GlcNAc deacetylases targets the prey bdelloplast peptidoglycan for destruction at the end of the predatory cycle, and without it a “ghost” of prey- bacterial peptidoglycan and some membrane remains after *Bdellovibrio* predation. The structure of the Bd3279 enzyme was determined and found to contain a deacetylase domain capped by a novel extra domain which may control access to the active site. Combining the double deletions of ∆*bd3279bd0468* with double deletions of predatory *dacB* genes *(∆bd3459bd0816*) resulted in a quadruple *Bdellovibrio* mutant which left behind a ghost of the same shape as the original prey bacterium upon completion of the predatory cycle. Such “hollowed out” bacterial ghosts can be prepared from diverse Gram-negative bacterial species and could have future applications in synthetic biology.

## Results

**Figure 1:**
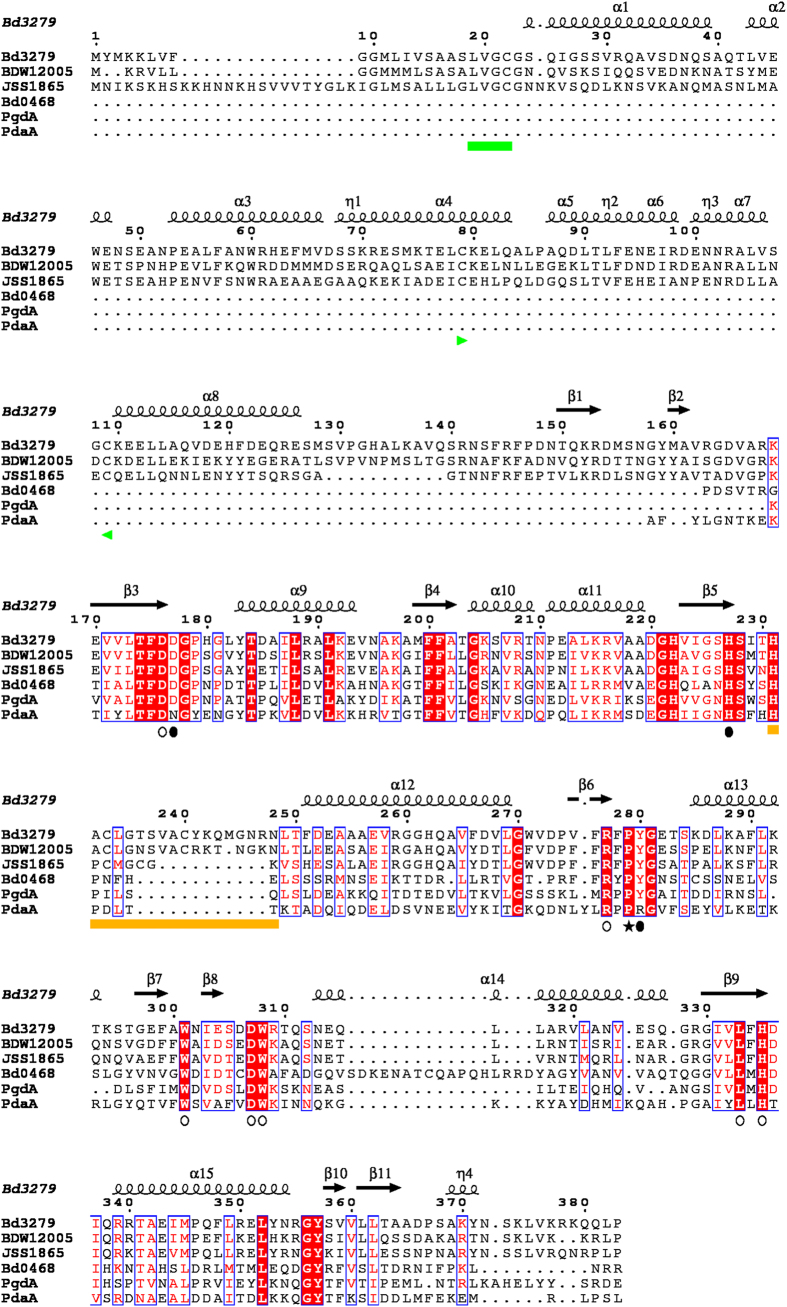
Multiple sequence alignment of peptidoglycan deacetylase domains of selected peptidoglycan N-deacetylases with secondary structure elements of Bd3279. PgdA = *S. pneumoniae* R6 PgdA; PdaA = *B. subtilis* 168 PdaA; Bd0468/Bd3279 = *B. bacteriovorus* HD100 Bd0468 and Bd3279; BDW12005 = *B. bacteriovorus* strain W Bd3279 homologue; JSS1865 = *Bdellovibrio exovorus JSS* Bd3279 homologue. Conserved metal binding residues are indicated by filled ellipses, conserved substrate binding residues are indicated by open ellipses, putative α-hydroxy-proline denoted with star, lipobox colored with green band, disordered active site loop colored with orange band. Sequences were aligned using ClustalW, and the output generated using ESPript^47^.

**Figure 2:**
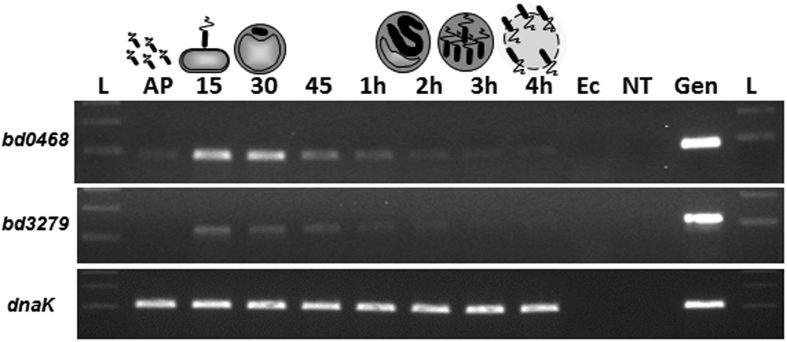
Reverse Transcriptase PCR showing the expression of potential GlcNAc N -deacetylase genes *bd0468* and *bd3279* and the known constitutive control gene *dnaK* over the predatory cycle of *Bdellovibrio bacteriovorus*. RNA was isolated at the time points indicated across the top of the gel during one round of synchronous *Bdellovibrio* infection of *E. coli* cells. Primers were designed to anneal specifically to the gene of interest. L = 100 bp DNA ladder, AP = Attack Phase cells, 15–45, 1 h–4 h = minutes or hours respectively since infection. Ec = *E. coli* strain S17-1 RNA (negative control: no *Bdellovibrio*); NT = control with no template RNA; gen = *B. bacteriovorus* HD100 genomic DNA (positive control). Expression of both deacetylase genes peaked at 15–30 minutes early in the predatory cycle while the positive control gene *dnaK* was expressed at all stages of the predatory cycle.

**Figure 3:**
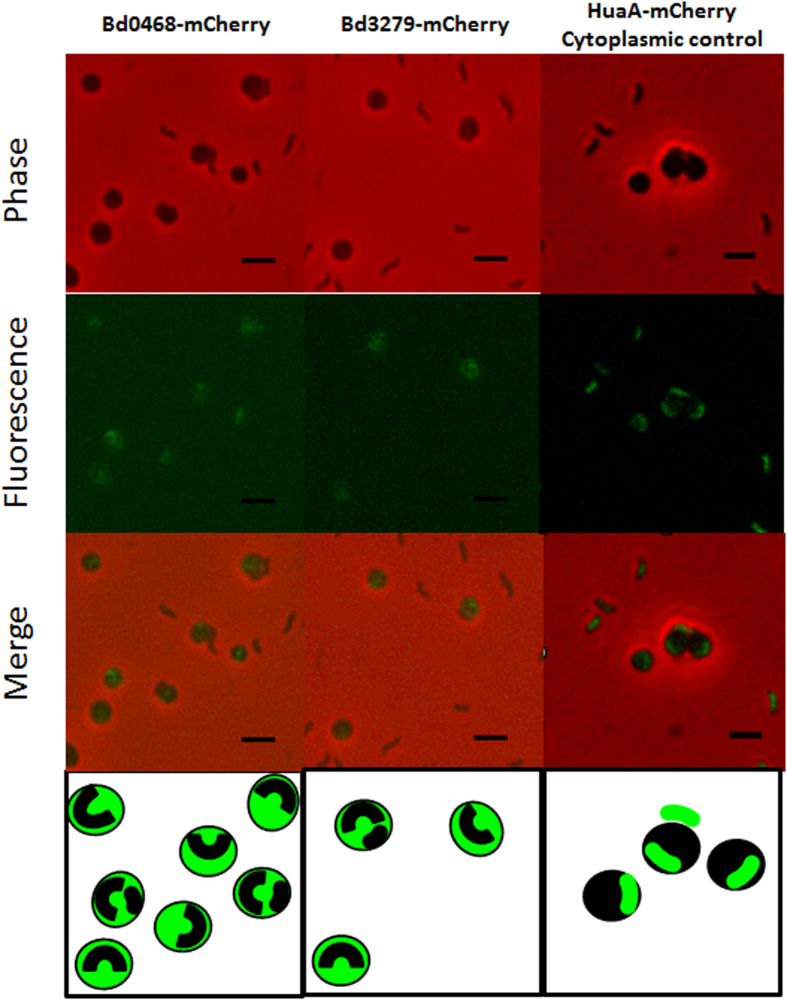
Epifluorescent images of wild type *B. bacteriovorus* HD100 expressing and secreting C-terminally mCherry tagged Bd0468 or Bd3279 proteins whilst invading non-fluorescent *E. coli* at 45 minutes post-mixing. Fluorescence was acquired with a two second exposure and maximum sensitivity gain. Despite the low intensity from the fluorescent tags on both proteins, they were clearly localised to the prey-bdelloplasts, with no tagged protein visible in the *Bdellovibrio* cells. Within the bdelloplasts, the outline of the invaded *Bdellovibrio* cell could be seen as a black backlit shadow against an mCherry background, indicating that the protein was exported into the bdelloplast periplasm and/or cytoplasm. Scale bars are 2 μm.

**Figure 4:**
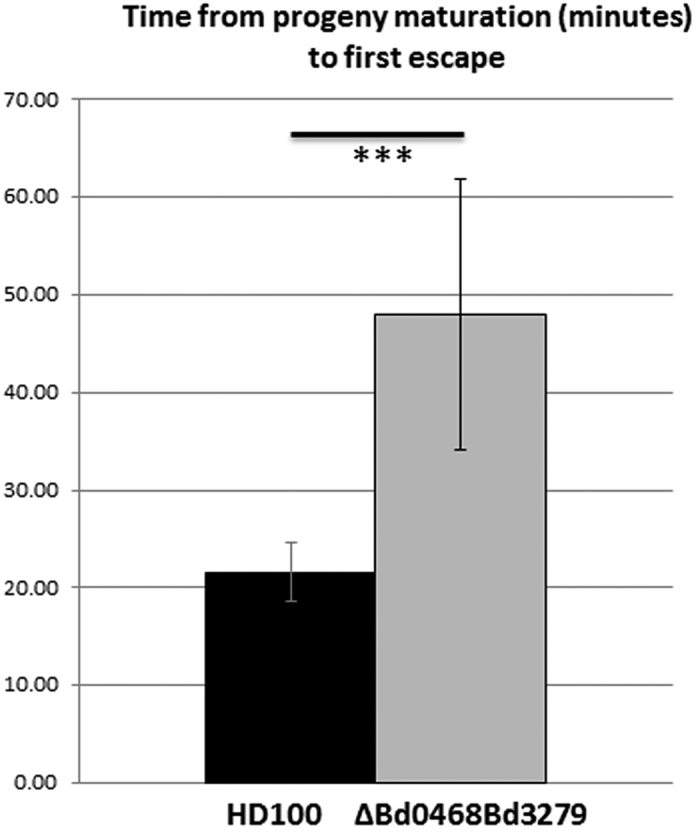
Histogram to show the time taken for progeny to escape from the prey cell as monitored by time-lapse phase contrast microscopy from at least three independent experiments. Time taken between progeny maturation and first progeny cell escape from the bdelloplast on an agarose surface was measured. HD100 = *B. bacteriovorus* wild type HD100; ∆Bd0468Bd3279 = double deacetylase mutant strain. Error bars are 95% confidence intervals. ***Student’s t-test p < 0.001.

We noted the homology between the predicted products of genes *bd0468* and *bd3279* and conservation of key metal binding residues important to known GlcNAc deacetylases of streptococci (Fig. 1)^6^. These genes are conserved in all sequenced *Bdellovibrio* strains and one homologue is present in the genome of the closely related, predatory *Halobacteriovorax*^7^ (see Supplementary Table S1). As transcription of the *bd0468* and *bd3279* genes peaked sharply at the point of prey-bacterial invasion by *B. bacteriovorus* HD100, but then declined during predatory growth inside bacteria (Fig. 2), we reasoned that the gene products were deacetylating the prey (or possibly predator) peptidoglycan during early stages of predation. Several repeat experiments showed that there was some low level expression of both genes throughout the predatory cycle, but that expression of both genes was mostly induced at 15–45 minutes post-mixing of predator and prey. Fluorescent tagging of the deacetylases with an mCherry fusion in the *Bdellovibrio* genome showed that each tagged protein was exported by the wild type predator into the prey bdelloplast (with external mCherry fluorescence backlighting the darker predator cells; Fig. 3). This suggests that the deacetylation activity was modifying the prey peptidoglycan, in agreement with other studies identifying Bd3279 as part of the *Bdellovibrio* secretome^8^. The fluorescent signal was weak implying that only low concentrations of the deacetylase proteins are required for prey-deacetylation. Induced expression of the *bd3279* and *bd0468* genes from plasmids inside *E. coli* led to damage of these cells (data not shown), so low level expression and export of the deacetylases is likely to be tightly controlled by the invading *Bdellovibrio*. In order to ascertain the function of the *bd0468* and *bd3279* gene products, a double deacetylase mutant (*∆bd0468bd3279*) was created. *B. bacteriovorus ∆bd0468bd3279* was still predatory, however progeny *Bdellovibrio* were released more slowly from prey bdelloplasts compared to wild type (Fig. 4) as determined by measuring the time from progeny maturation as the growing, long intraperiplasmic *Bdellovibrio* divides into multiple shorter progeny (seen by fluorescent backlighting of the prey periplasm) to the time that the first progeny *Bdellovibrio* leaves the outer layers of the prey. A minimum of 2 biological repeats and n > 30 were carried out and Student’s T-test gave a *p* value of 2.6 × 10^−8^, demonstrating significance. This extended the time taken for completion of predation, with some bdelloplasts still seen at 4 hours post-mixing of double deacetylase predator and prey (very few were seen in predation by wild type predators at this timepoint), but virtually no bdelloplasts seen at 5 hours post-mixing. Despite this, a standard predatory culture of the mutant had cleared of prey overnight similar to wild type.

**Figure 5:**
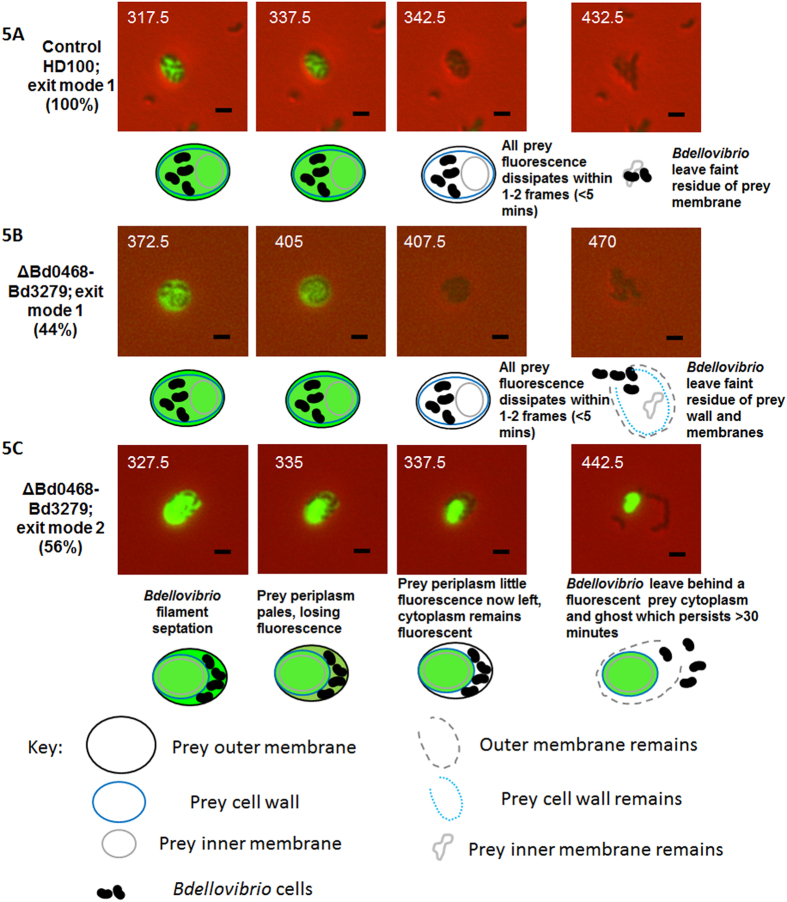
Epifluorescence phase contrast microscopy of *Bdellovibrio* (small, phase dark, comma-shaped cells) preying upon *E. coli* prey cells which have periplasms constitutively fluorescently labelled by a pMal::mCherry fusion (and cytoplasm labelled as the fusion protein is expressed). The images are from late in the predation cycle, with time post-mixing of predator and prey shown in minutes to the left of each image. With wild type *B. bacteriovorus* HD100 (**A**) and in 44% of the double mutant (**B**), total bdelloplast fluorescence is lost within 2 frames (<5 min; exit mode 1) as the inner membrane is breached by exiting predator. 56% of the double ΔBd0468Bd3279 mutants (**C**) display a different pattern, with fluorescence lost from the prey periplasm within 2 frames, but retaining fluorescence in the original prey cytoplasm compartment even after the *Bdellovibrio* cells (which in this case have grown between wall and outer membrane only), have become motile and broken out of the bdelloplast; (exit mode 2). Scale bars are 1 μm.

Using prey *E. coli* S17-1 expressing maltose-binding protein tagged with mCherry, we visualised fluorescent cytoplasmic and periplasmic compartments of the prey during predation by non-labelled *B. bacteriovorus ∆bd0468bd3279* versus wild type *B. bacteriovorus* HD100 (Fig. 5). Wild type *B. bacteriovorus* HD100 completed predatory replication in the periplasm of the prey cells, in the compartment between sacculus and inner membrane (Fig. 5A) with no substantial fluorescent prey material remaining after prey bdelloplast lysis. However the *B. bacteriovorus ∆bd0468bd3279* mutant displayed two different prey-exit behaviours indicating an ability to occupy and thus later leave different periplasmic compartments- inside or outside the prey bacterial wall. A minority of the *∆bd0468bd3279* mutant population (44%; see methods) exited the fluorescent prey in a partly similar way to the wild type (Fig. 5B), whereby no fluorescent prey material was left behind, although in contrast to wild type, a noticeably round translucent prey structure remained.

**Figure 6:**
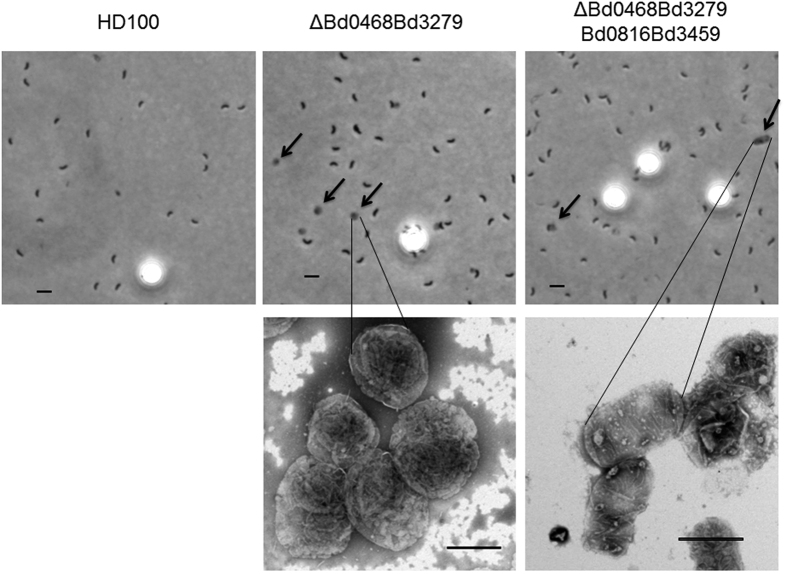
Phase contrast (top) and transmission electron (lower) microscopy showing residual, phase-light, prey “ghost” structures (arrows) prevalent in the culture medium of mutant *Bdellovibrio* after 24 h of prey lysis. Smaller phase dark *Bdellovibrio* cells and larger phase bright beads used to control for enumerations of the prey ghosts can also be seen in the phase contrast images. The ghost structures left behind in the quadruple mutant were more rod-shaped as the absence of the two predatory *dacB* gene products meant that bdelloplasts resulting in predation by this strain were not rounded up. Much fewer were seen in the HD100 cultures, but smaller vesicles were sometimes seen by electron microscopy that were barely visible by phase contrast microscopy. Scale bars are 1 μm.

**Figure 7:**
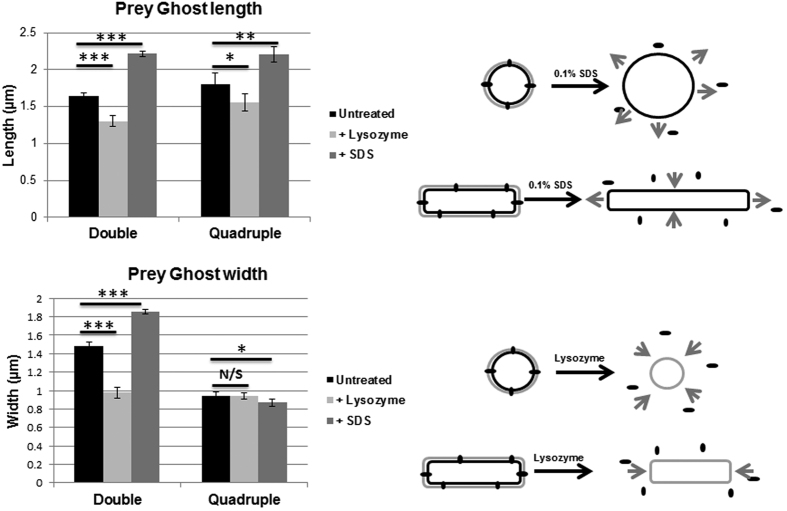
Average length and width of “ghost” residues left behind after predation by *Bdellovibrio* deacetylase and endopeptidase mutants. Purified “ghost” residues were imaged by electron microscopy with 2%PTA staining and sizes measured using Simple PCI software. Samples of ghosts were treated with final concentrations of 0.1% SDS (+SDS) or 100 μgml^−1^ lysozyme/10 mM EDTA (+lysozyme). Double = ∆Bd0468∆Bd3279 mutant strain, Quadruple = ∆Bd0468∆Bd3279∆Bd0816∆Bd3459 mutant strain. Error bars are 95% confidence intervals, ***Student’s t-test p < 0.001; **Student’s t-test p < 0.005; *Student’s t-test p < 0.05. n = 25 to 325. Treatment by SDS resulted in expansion of width and length for the double mutant, and expansion of length but slight reduction of width for the quadruple mutant. Treatment by lysozyme reduced length and width for the double mutant, but only reduced length of the quadruple mutant. The schematic diagram represents this.

A greater proportion of the *∆bd0468bd3279* mutant population (56%; Fig. 5C) completed predation in an outer periplasmic layer and exited through the prey outer-membrane, leaving an intact, fluorescent, ovoid-round, prey-structure, comprising (as we confirm in later data Figures) the sacculus, inner membrane and cytoplasmic components. We deduce this mutant behaviour to be *Bdellovibrio* replication *on the outer surface* of the prey sacculus in the periplasm, in the compartment between sacculus and outer-membrane. Microscopically, the dark *Bdellovibrio* progeny cells, prior to escape by this 56% of the population, were seen pressed in a thin layer around the periphery of the fluorescent *E. coli* periplasm, unlike the more central position of the wild type predators or the remaining 44% of the mutant population (Fig. 5A,B). The *E. coli* prey-derived structures remaining after exit by the mutant *Bdellovibrio* population were termed “prey ghosts” and were seen abundantly persisting alongside newly released *Bdellovibrio* progeny in the deacetylase mutant predatory cultures, but not in wild type predatory cultures. The ghosts were visible as faintly semi-translucent structures by phase contrast microscopy, and in more detail with PTA staining and electron-microscopy (Fig. 6). They persist in the medium of predatory cultures of *B. bacteriovorus ∆bd0468bd3279,* many hours after *E. coli* prey lysis. Such large, abundant prey ghosts are not visible in the cultures of wild type *Bdellovibrio* that have preyed upon *E. coli,* only smaller traces of degraded or folded prey material are seen. The prey ghosts remaining after predation of *E. coli* S17-1 with *B. bacteriovorus ∆bd0468bd3279* were ovoid, with mean measurements of 1.64 ± 0.31 μm by 1.48 ± 0.26 μm (n = 81) (Figs 6 and 7). These ovoid dimensions are similar to that of bdelloplasts, which, during predation by wild type *Bdellovibrio* are rounded by the action of two *dacB* gene products on the prey peptidoglycan^1^.

### Composition of prey ghosts

**Figure 8:**
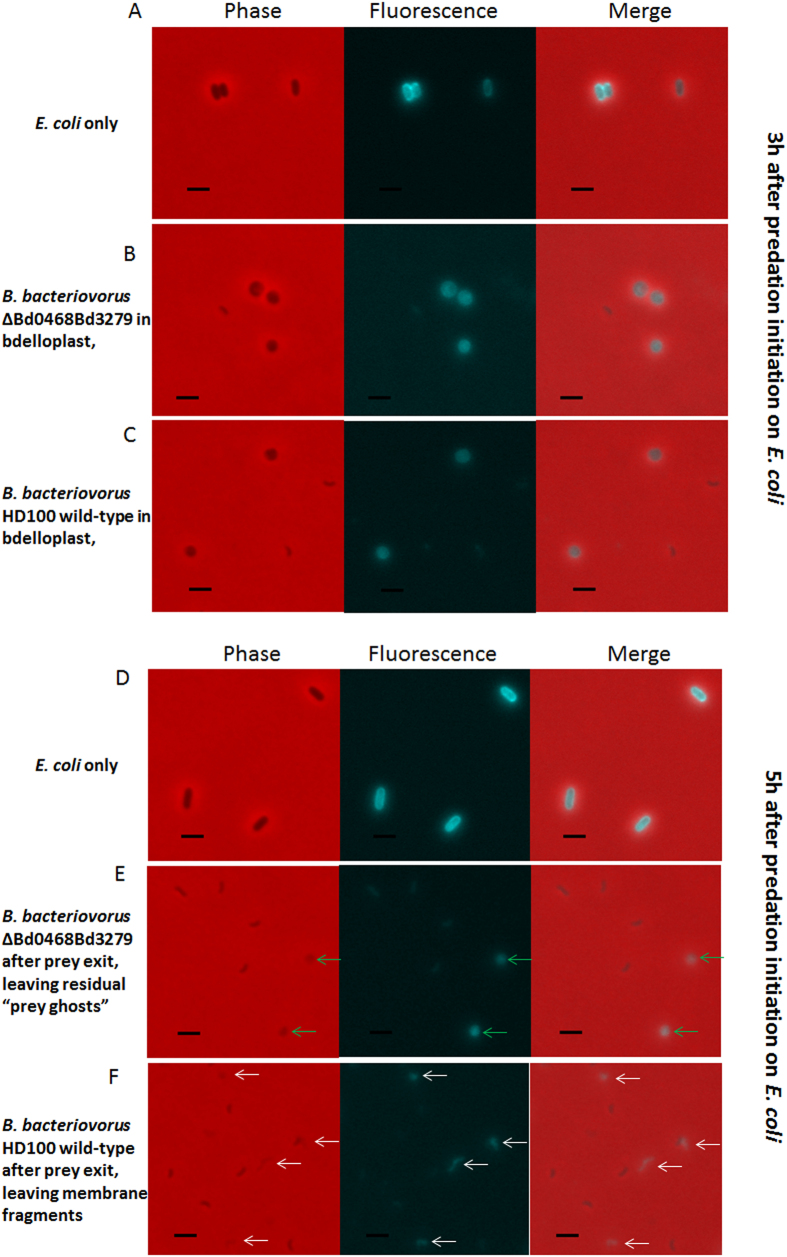
Epifluorescence phase contrast microscopy of TMA-DPH-stained cultures of *Bdellovibrio* (small, phase dark, comma-shaped cells) preying upon *E. coli* prey. 10 mM of the fluorescent, membrane-staining dye TMA-DPH was used in 2 minute staining reactions at the timepoints described. **(A)** Uninfected *E. coli* control cells after 3 hours incubation in Ca/HEPES buffer **(B)** *Bdellovibrio* ∆Bd0468Bd3279 at a control timepoint of 3 hours after invading and rounding *E. coli* “bdelloplasts”. Also visible is a smaller, less fluorescent *Bdellovibrio* attack phase cell. **(C)** Wild type *Bdellovibrio* at a control timepoint of 3 hours after invading and rounding *E. coli* “bdelloplasts”. Also visible is are smaller, less fluorescent *Bdellovibrio* attack phase cells. **(D)** Uninfected *E. coli* control cells after 5 hours incubation in Ca/HEPES buffer **(E)** Lysed bdelloplasts 5 hours post mixing, invasion, predation and exit of *Bdellovibrio* ∆Bd0468∆Bd3279 into/from prey *E. coli* shows fluorescent, rounded prey ghosts of similar size to the bdelloplasts (in **B**,**C**), but paler under phase contrast. **(F)** Lysed bdelloplasts 5 hours post mixing, invasion, predation and exit of *Bdellovibrio* wild type *Bdellovibrio* HD100 strain into/from *E. coli*. Here, smaller, round, fluorescent structures or fragments of prey are visible in the fluorescent channel, but scarcely visible in the phase contrast images. Scale bars are 2 μm.

The prey ghosts stained internally and externally with the membrane-specific stain TMA-DPH indicating that they still comprised of substantial amounts of membrane material (Fig. 8E) in addition to remaining prey peptidoglycan (see below) that supported their rounded structure. A TMA-DPH-staining control of predatory cultures (wild type *Bdellovibrio* preying upon *E. coli*) revealed, at low frequency (Fig. 8F) much smaller round or fragmented pieces of membrane. These most likely represent remains of the inner membrane, as the wild type *Bdellovibrio* completely digest the prey peptidoglycan, with collateral effects physically disintegrating the prey outer membrane into smaller fragments upon prey lysis, allowing predator exit^4^.

**Figure 9:**
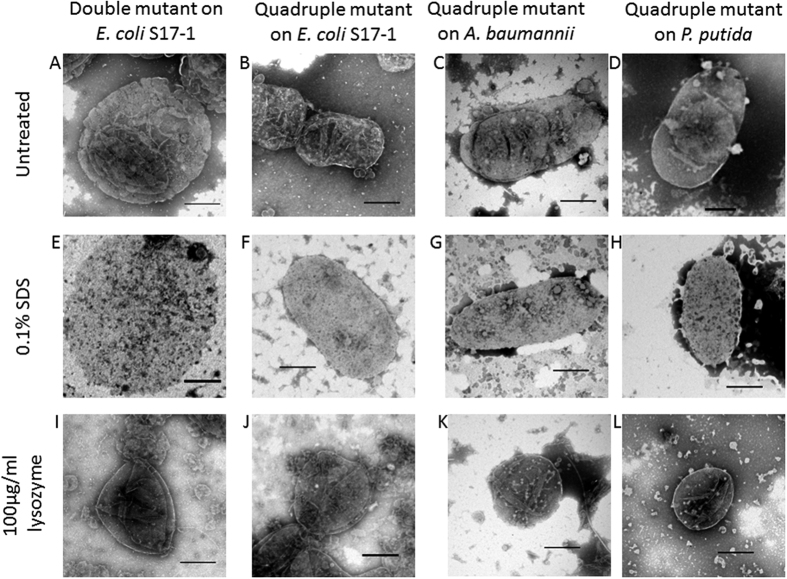
Transmission electron micrographs showing prey ghost structures from different bacteria after 24 h predation by mutant *Bdellovibrio*. Samples of ghosts were treated with final concentrations of 0.1% SDS or 100 μgml^−1^ lysozyme/10 mM EDTA. Double = ∆*bd0468*∆*bd3279* mutant strain, Quadruple = ∆*bd0468*∆*bd3279*∆*bd0816*∆*bd3459* mutant strain. *E* = *Escherichia, A* = *Acinetobacter, P* = *Pseudomonas.* Scale bars are 500 nm.

**Figure 10:**
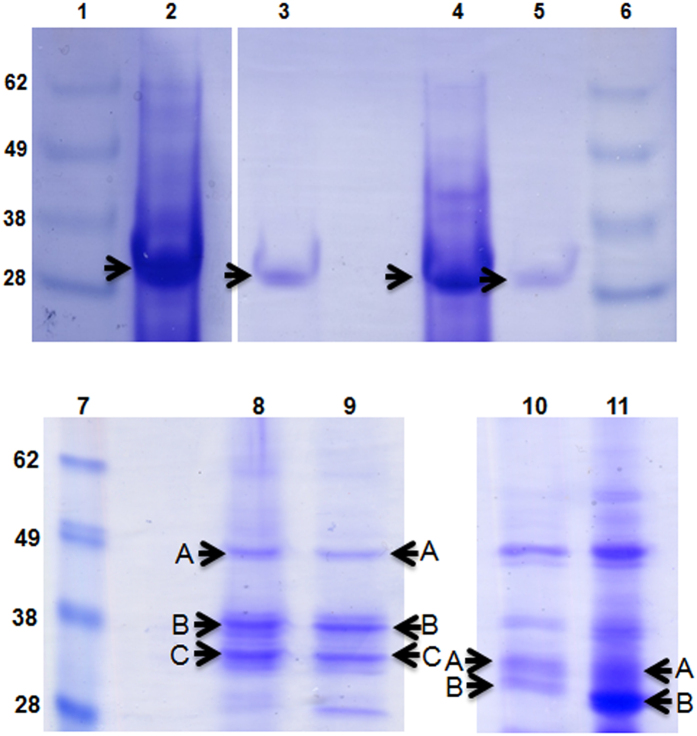
SDS-PAGE analysis of purified prey ghosts. The protein bands indicated by the arrows were extracted and subjected to mass spectrometry. Lanes 1, 6 and 7- Molecular weight marker; sizes are indicated in KDa. Prey ghost samples for the other lanes were generated using *Bdellovibrio* mutants and prey type indicated in Table 1. Highlighted protein bands were analysed by mass spectrometry, the resulting top hits using Mascot are indicated in Table 1. Prey ghosts from predation by the wild type HD100 strain were not numerous or stable enough to analyse by SDS-PAGE.

To verify that the prey ghosts we observed contained peptidoglycan, we combined the deletion of two known *B. bacteriovorus dacB* genes (*bd3459, bd0816*) encoding cell wall active DD-endopeptidases (which are responsible for decrosslinking prey PG and rounding prey bdelloplasts) with the double deacetylase deletion to produce a “quadruple mutant”: *B. bacteriovorus ∆bd0468bd3279bd0816bd3459*. We reasoned that adding in this extra double mutation would demonstrate whether the ghost structures contained prey peptidoglycan, as only that target is acted upon by the DacB enzymes rounding the prey in wild type bdelloplast formation^1^. These predatory DacB enzymes cut higher order cross-links, but the action is limited to softening the prey cell wall rather than resulting in lysis. Deleting the *dacB* genes from the already double deacetylase mutant *Bdellovibrio* would therefore abolish that rounding capacity and the shape difference would confirm peptidoglycan as a major ghost component. Predation of *E. coli* with the quadruple mutant *Bdellovibrio* resulted in bacterial-shaped prey ghosts in the culture medium, rather than the ovoid prey ghosts produced by the double deacetylase mutant that retains wild type rounding capability (Fig. 9b versus **9a**). This suggests (as one would expect), that the deacetylation of prey PG has no effect on it as a target for predator DD-endopeptidase activity which results in rounding of the prey wall; rather it affects predator peptidoglycan-glycosylase activity at the end of predation which degrades the remainder of the wall in wild type predation. At this late point in the predation cycle, the prey is long dead and its contents degraded and used by *Bdellovibrio* for predatory growth, so autolysis by remaining prey enzymes is unlikely. Prey ghosts in Fig. 9 and Supplementary Figure S2 were purified away from *Bdellovibrio* cells, by differential centrifugation on Percoll gradients (41.4–49.4% Percoll in 0.3 M NaCl), followed by a subsequent wash in distilled water (Supplementary Figure S3). This simple isolation protocol gave relatively pure preparations of prey ghosts with few contaminating uninfected prey or predator cells, which appeared unchanged after incubation at 4 °C for up to 2 weeks or stored at −20 °C for several months. Predation with the quadruple mutant also gave native bacterial-shaped ghosts for prey cells of *Acinetobacter baumannii, Pseudomonas putida, Proteus mirabilis* and *E. coli* 083:H1 (Fig. 9 and further examples in Supplementary Figure S2). Each of these prey ghosts contained a central deflated vesicle-like structure, which we propose (bearing in mind the TMA-DPH membrane-staining of ghosts in Fig. 8E) is the residual cytoplasmic membrane (remaining after cytoplasmic contents have been consumed by *Bdellovibrio*). When the protein content of these ghosts was examined by SDS-PAGE and MALDI-QTOF mass spectrometry, the dominant proteins identified were outer membrane porins (OMP) of the prey (Fig. 10 and Table 1). It is interesting to note that all of these bands examined were smaller than the predicted size of the OMP. This could be the result of cleavage of a signal peptide (which should result in ~3 kDa smaller predicted size) combined with anomalous running in the gel due to the hydrophobic nature of these proteins, or it could indicate that some degradation of these OMPs has occurred in the prey ghosts. There are older reports^9^ of internalised *Bdellovibrio* altering bdelloplast permeability; OMP partial degradation could be a mechanism for this, but testing this is beyond the scope of this study. These SDS-PAGE results indicate that the ghost-structures retain an (at least partial) outer-membrane in addition to the prey cell wall. Electron micrographs of untreated prey ghosts (Fig. 9A–D) and cytoplasmic fluorescence retention seen in Fig. 5 also suggests an inner membrane structure remains inside the wall in the prey ghosts. If the prey ghosts also retain such an inner membrane similar to vesicles solely seen left over from wild type predation by TMA-DPH staining (Fig. 8E), then it is likely deproteinated by the *Bdellovibrio* proteases as no inner membrane proteins were detected.

**Figure 11:**
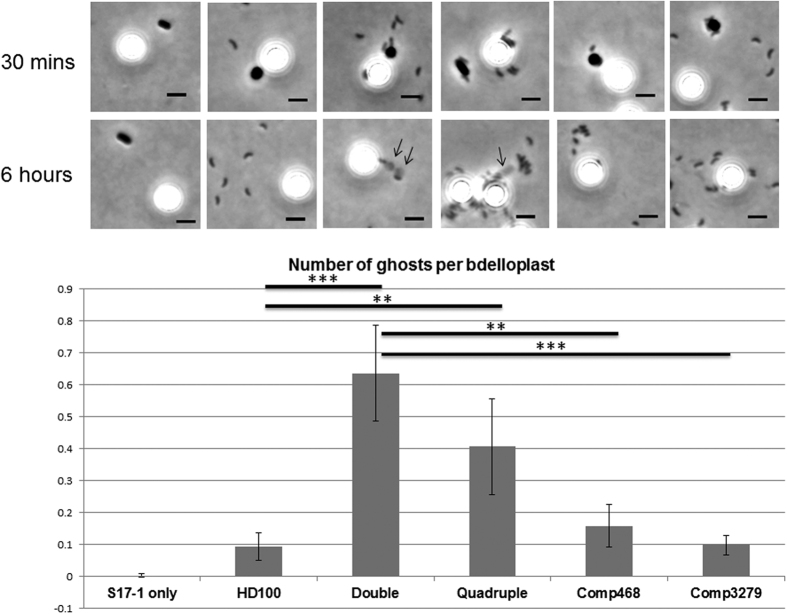
Number of ghosts per bdelloplast for each strain shows complementation of the double mutant phenotype by either of the deacetylase genes. Synchronously growing predatory cultures were analysed by phase contrast microscopy and scored for number of bdelloplast structures (phase dark spheres, numbers were compared to mixed-in beads as control: the larger, phase bright objects seen) at 30 minutes and ghost structures (lighter grey structures indicated by arrows) at 6 hours. S17-1 only = *E. coli* S17-1 prey cells without *Bdellovibrio* added, Double = ∆Bd0468∆Bd3279 mutant strain, Quadruple = ∆Bd0468∆Bd3279∆Bd0816∆Bd3459 mutant strain, Comp468 and Comp3279 are the double mutant complemented with *bd0468* or *bd3279* respectively. Error bars are 95% confidence intervals. ***Student’s t-test p < 0.001; **Student’s t-test p < 0.005

To further examine the structure of the prey ghosts, we treated them with lysozyme to digest the peptidoglycan, or SDS to solubilise the lipids into micelles and disconnect lipoprotein or membrane protein interactions. Treatment of the prey ghosts from predation by the quadruple *Bdellovibrio* mutant with 0.1% SDS (Fig. 9E–H) or with hen egg white lysozyme (Fig. 9I–L) changed their shape, size and/or appearance. SDS treatment altered the surface appearance of the prey ghosts, removing a rippled feature from the surface (likely the OM), and leaving a more granular structure (peptidoglycan with a surface resembling a concrete breeze block); the internal, deflated, putative-cytoplasmic membrane vesicle was also no longer visible. SDS treatment also increased the size in all dimensions of prey ghosts produced by the double deacetylase mutant, whereas with prey ghosts from the quadruple mutant, the length increased while the width decreased (Fig. 7). Lysozyme treatment removed the bacterial-shaped and sized ghost structure, leaving solely a vesicle the size, or slightly larger, of the deflated putative cytoplasmic membrane vesicles seen within the untreated prey ghosts (Fig. 9I–L and Supplementary Figure S2a1–3). Such prey-vesicles (without peptidoglycan) were scarcely visible by phase contrast microscopy and so have not been studied previously, but it may be that a deflated cytoplasmic membrane vesicle (only) is a normal remnant of wild type predation, but was only noticed entrapped in the ghosts after predation by deacetylase mutant *Bdellovibrio*. Membrane vesicles (similar to those liberated by lysozyme treatment from the prey-ghosts) were indeed observed, by staining with TMA-DPH membrane-stain, in predatory cultures of wild type *Bdellovibrio* that have consumed and lysed bacterial prey (Fig. 8F). Effective complementation of the double deacetylase mutant was achieved by conjugating either of the two genes (*bd0468* or *bd3279*) back into the double mutant, significantly reducing prey-ghost-like structures to wild type levels (Fig. 11). This, together with the fact that no prey ghosts were seen in the single mutants, suggests redundancy of function, with either products of *bd0468* or *bd3279* capable of enough deacetylation of the prey peptidoglycan to mark it for destruction at the end of the predatory lifecycle. It is likely that these enzymes have some affinity differences to different peptidoglycan (*Bdellovibrio* has a broad prey range) and this redundancy is common in *Bdellovibrio,* with multiple genes for the same function^1^,^10^,^11^.

Rotem and coworkers^12^ describe that prey ghost envelopes of cultured *E. coli* cells (without predators present) obtained by osmotic shock are attached to and invaded by attack phase *Bdellovibrio,* but these are unlike the predator-produced prey ghosts in our work. Our time-lapse microscopy demonstrated that in the vast majority of cases, attack phase *Bdellovibrio* attached and then detached from prey ghosts left by the double deacetylase mutant (only one example of entry into a prey ghost-like structure was seen from 16 fields of view of 10–50 prey ghosts in each field from 3 biological repeats; data not shown), suggesting that the ghost envelope was modified or degraded enough that it was no longer recognised as suitable prey for *Bdellovibrio*. The prey ghost envelopes obtained by osmotic shock are likely much less modified and therefore are recognised as suitable prey for invading *Bdellovibrio* as they likely retain relatively unchanged outer cell layers. The fact that *Bdellovibrio* entered the prey ghosts derived from the deacetylase mutant only very rarely suggests that the outer layers of these are modified to an extent where most of the material is no longer recognised by the *Bdellovibrio* as a suitable prey cell. This is logical as they are exhausted of usable materials by prior predation.

**Figure 12:**
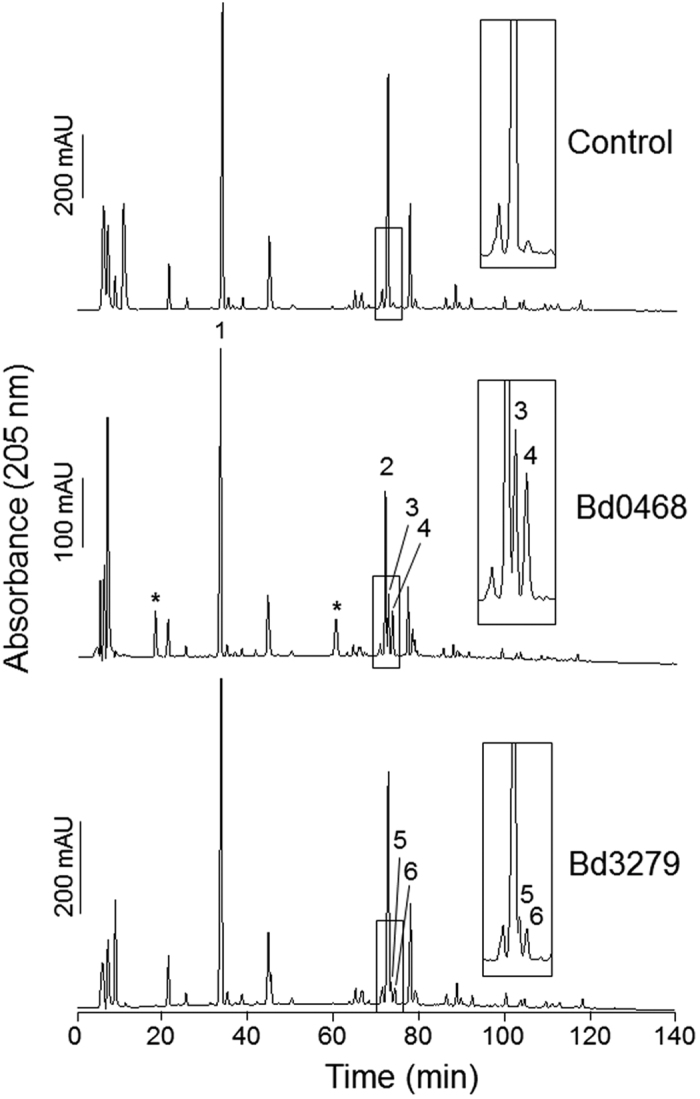
HPLC chromatogram showing muropeptide analysis of *E. coli* D456 PG which had been treated with Bd0468, Bd3279 or buffer (control). New peaks can be detected (numbered 3–6) that are not present in the control. *peaks derived from purified Bd0468 protein solution. Peaks 1–6 were analysed by MS/MS (Table 2) which showed that Bd0468 and Bd3279 are peptidoglycan GlcNAc deacetylases.

To verify the activity of the deacetylase enzymes, they were heterologously expressed, purified and tested against isolated peptidoglycan sacculi from *E. coli* D456 (prepared conventionally) followed by digestion with the muramidase Cellosyl and analysis of the resulting muropeptides by HPLC. As seen in Fig. 12, extra peaks were observed in the treated peptidoglycan relative to the untreated control. The additional peaks numbered in Fig. 12 were investigated by using linear quadrupole ion trap with combined Fourier transform ion cyclotron resonance mass spectrometry (LTQ-FT MS); the results of which are shown in Table 2. This analysis revealed ionized peptidoglycan peptides lacking masses corresponding to the lack of one (42 Da) or two (84 Da) acetate groups relative to 1864 Da which is the fully acetylated TetraTetra dimer; and further analysis of the product peaks by tandem MS confirmed that both Bd0468 and Bd3279 are GlcNAc-specific peptidoglycan deacetylases. We could not identify a deacetylated version of the main uncross-linked (monomeric) peak, Tetra. Either the enzymes were specific for cross-linked peptidoglycan, or uncross-linked peptidoglycan is a substrate resulting in the deacetylated Tetra co-eluting with the monomeric Tetra.

### Domain-structure of the representative deacetylase Bd3279

**Figure 13:**
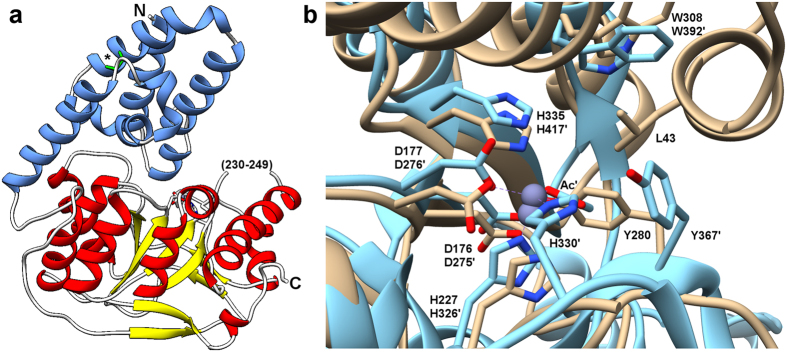
Structure of *Bdellovibrio* peptidoglycan deacetylase Bd3279. (**a)** Protein fold of Bd3279, N-terminal domain coloured in blue, C-terminal catalytic domain coloured by secondary structure (TIM barrel; red α-helices, yellow β-strands). The N and C termini and disordered active site loop (parentheses) are labelled. Disulphide link between C79 and C109 is shown in stick form (labelled by asterisk) and coloured green. (**b)** Comparison of selected active site residues of Bd3279 and *S. pneumoniae* PgdA (PDB code 2C1G). Bd3279 (light tan, non-primed labels) and PgdA (light blue, prime labels) have a largely similar active site conformation, centred around a catalytic Zn ion (purple, sphere representation). Corresponding residues on the left-hand side of the figure (toward the core of the enzyme) are in better agreement than those on the right-hand side (towards solvent and the disordered loop between residues 230–249); the Bd3279 equivalent to PgdA H330’ (H231) is within this region and not observed in our structure. The conserved active site tyrosine (Y280/Y367’) is adjacent to this region and in Bd3279 takes the place of the PgdA substrate-mimic acetate ligand (labeled Ac’). The figure also demonstrates the extent of which the N-terminal domain “caps” the active site pocket – in particular, L43 contacts both H335 and Y280.

We obtained a high-resolution structure of the secreted, mature form of Bd3279 (encompassing amino acids 24–383) at 1.5 Å resolution (Fig. 13). Bd3279 is comprised of two domains, an N-terminal domain (NTD, amino acids 24–126) and the catalytic C-terminal domain (CTD, amino acids 127–383). The NTD forms a compact structure comprised of seven regular and two 3_10_ α-helices, stabilized by a disulphide bond between residues Cys79 and Cys109. This domain approximately resembles a “cap” over the active site of the enzyme, and has no precedent in existing peptidoglycan deacetylase protein structures; indeed, the sequence of this region appears to be unique to *Bdellovibrio* and its close relatives.

The region of the NTD formed by residues 33–52 protrudes into the “cracked” region of the catalytic barrel (the strands that are lacking helices on their external edges), contributing a wing-like cover to the active site cleft. The remaining helices of the NTD form a helical bundle that contacts the loop regions from the last two α/β units of the CTD. The overall NTD fold has closest structural similarity to a broad variety of helical bundle protein domains – *e.g.* DALI Z-score/RMSD of 6.2/2.3 with a region of the *Saccharomyces cerevisae* proteosome β1 subunit^13^; 5.6/3.2 for a glycosylhydrolase inhibitor protein from *Streptococcus pyogenes*^14^ if the helix abutting the CTD is included (residues 109–126)). The hinge between the NTD and CTD is formed by a region with no classical secondary structure (residues ~128–148) that packs against the edge and underside of the barrel.

The catalytic CTD shares significant sequence and structural homology with those from other characterized CE-4 (carbohydrate esterase 4) superfamily members – *e.g.* DALI^15^ Z-score/RMSD of 22.7/2.2 with an *Aspergilus nidulans* chitin deacetylase (PDB 2Y8U, unpublished); 21.4/2.2 with the *Streptococcus pneumoniae* PgdA peptidoglycan deacetylase^6^. This structural agreement is strongest in the core of the CTD (residues 169–360). One of the active site loops of Bd3279 is at least partially disordered, with weak electron density for amino acids 229/230 and no interpretable electron density for region 231–248. This presumed flexible loop contains two cysteine residues seemingly unique to Bd3279 and its closest predatory bacterial homologues (the equivalent region in other peptidoglycan deacetylases is short, e.g. 5 amino acid residues in PgdA).

### Active site features of Bd3279

The active site of Bd3279 comprises the canonical conserved amino acids of the CE-4 superfamily^16^, and also those particular to the peptidoglycan deacetylase subgroup. The CE-4 ×^2+^ catalytic ion binding pocket is present in Bd3279, co-ordinated by residues His227 and Asp177; the third superfamily-conserved ion liganding residue would be expected to be contributed by His231, which is present in the disordered active site loop and thus not modelled in our structure. In-line with homologous CE-4 domains, we have modelled the ion as Zn^2+^, putatively arising from co-purification as no exogenous zinc was added at any stage. The Zn^2+^ ion interacts with the OH group of the nearby Tyr280 (2.3 Å), analogous to the interaction observed for PgdA with a substrate mimic acetate oxygen atom^6^. The residues of PgdA responsible for interaction with substrate (or aligning side chains that directly interact with substrate) are conserved in Bd3279–Asp176, Arg277, Trp301, Asp307, Trp308, Leu333 and His335 are in a similar conformation to PgdA Asp275 (catalytic base), Arg364, Trp385, Asp391, Trp392, Leu415 and His417 (catalytic acid), respectively. We observe strong density at the Cα atom of the conserved Pro279, a feature observed in other characterized peptidoglycan deacetylases, BC0361 from *Bacillus cereus* and BA0330 from *B. anthracis*^17^,^18^. In agreement with these studies we have modelled the density as an α-hydroxy-L-proline species. Unique to Bd3279, the NTD sits over these active site motifs, with residues Leu43, Val44, Trp46 and Glu47 projecting into the substrate-binding cleft; such proximity may regulate enzyme activity. In total, the structure of Bd3279 confirms that deacetylase activity is likely to be attributable to the same conserved machinery of other, previously characterized CE-4 enzymes, but with unique flanking features (obscuring of substrate binding cleft by novel N-terminal domain “plugging”; extension of active site by disordered loop) that currently appear to be predator-specific.

## Discussion

During the investigation of the roles played by peptidoglycan deacetylases in predation by *B. bacteriovorus* HD100, we found that a double mutant (*∆bd0468bd3279*) lacking two such enzymes leaves behind a significant proportion of the prey cell after predation is complete, something not seen after predation with wild type *Bdellovibrio*. These rounded “prey ghosts” consist of peptidoglycan surrounded by outer membrane with some inner membrane remaining, but with the inner membrane denuded of proteins. On *further* deletion of two genes (*bd0816* and *bd3459*) encoding predatory DacB enzymes known to cause de-crosslinking of the wall^1^, the prey ghosts remaining after predation retained their initial shape (rod-shaped in the case of *E. coli*), experimentally verifying that the physically robust nature of the ghost structures was due to their peptidoglycan content.

The mCherry-fluorescent cytoplasm of *E. coli* prey cells was retained as spheres in buffer (Fig. 5C final timecourse image) by this ghost peptidoglycan after mutant predators, that replicated only between the prey sacculus and outer-membrane, exited through the outer membrane.

These prey ghosts, containing inner-membrane, cell wall and outer-membrane represent a serendipitous source of “bacterial skeletons” that could potentially be useful for synthetic biology as a starting point for the test insertion of artificial chromosomes in the bottom-up *de novo* construction of synthetic cells which can then autonomously sustain themselves and proliferate. Although there are multiple potential mechanisms for producing and dividing membrane-only-based synthetic cells^19^, these cells do not readily withstand osmotic pressure or environmental changes. Furthermore, L-form bacteria without cell walls are also osmotically unstable and tend to abandon their normal cell division machinery which efficiently divides the cell contents between daughter cells^20^. The prey ghosts discovered here could provide a convenient, pre-made test-chassis for synthetic cells, with at least partial inner and outer membranes that would not be present on conventionally isolated bacterial cell wall sacculi produced by the method of boiling with SDS^21^.

The fact that a stable peptidoglycan-containing prey ghost structure was produced in the *absence* of two predator GlcNAc deacetylase enzymes contradicted the suggestion of Thomashow and Rittenberg^3^ that such enzymes act to *stabilise* the prey peptidoglycan during predation. They had proposed^3^ that deacetylation of prey peptidoglycan protected the bdelloplast from premature lysis from an early-acting *Bdellovibrio* “glycanase” glycosylhydrolase activity (during predation); such a glycanase was suggested to be forming a limited entry pore in the prey wall for *Bdellovibrio* invasion. This hypothesis could still hold true for MurNAc deacetylation (and we are actively investigating genes for that process), however we have shown here that removal of two GlcNAc deacetylases did not result in premature lysis of prey, and so this is not the mechanism of limiting the early glycosylhydrolase activity. Actually, we have established that the prey peptidoglycan is deacetylated during predation but that this reaction makes it susceptible for a later destruction (by an enzyme(s) we are looking to discover) at the end of predation. Without that destruction, prey ghosts remain.

The isolation of the ghosts also allowed us to test a different long-held hypothesis in *Bdellovibrio* research; Thomashow and co-workers^3^ found in 1978 that *E. coli* bdelloplasts infected with wild type *B. bacteriovorus* 109J were resistant to (hen egg white) lysozyme and attributed this to *N*-deacetylation of the prey peptidoglycan. Our observation that the prey ghosts remaining, after *E. coli* predation with GlcNAc peptidoglycan deacetylase minus *B. bacteriovorus ∆bd0468bd3279,* were sensitive to lysozyme agrees with this.

Our observation that a significant proportion of the (*∆bd0468bd3279)* mutant predators entered the outer membrane of the prey, but did not pass all the way through the wall, suggests the opposite effect to that above: rather than reducing the actions of the initial pore-forming glycosylhydrolase enzymes the deacetylation instead may facilitate this process, allowing a higher percentage of wild type predators to traverse the prey wall. *B. bacteriovorus* has a wide prey-range including diverse genera of Gram-negative bacteria with widely differing wall compositions and so the degree to which they penetrate the outer layers of those different prey (beyond the *E. coli* used here) may vary considerably naturally. As peptidoglycan lysis occurs after predator septation, products liberated from prey wall breakdown will only be able to contribute to cytoplasmic nutrient stores in predators rather than allowing extra progeny to form.

The mutant *Bdellovibrio ∆bd0468bd3279* population were slower overall to exit prey than the wild type, likely due to the majority of the prey peptidoglycan not being degraded, and therefore acting as a barrier to slow exit of the exhausted prey bdelloplast. Clearly, in the case of the 44% subpopulation of mutant *Bdellovibrio ∆bd0468bd3279* which enter the sacculus, some peptidoglycan must be locally degraded for them to be released. This peptidoglycan could be at the same local area as the proposed entry pore^22^ made (and later resealed) as the *Bdellovibrio* entered through the sacculus; this therefore may be of a different composition to the rest of the sacculus. In electron micrographs of the prey ghosts, a discrete hole the size of the width of a single *Bdellovibrio* was sometimes observed (Fig. 10) especially in the SDS-treated samples. This supports the entry pore reuse hypothesis, as does the observation that all of the *Bdellovibrio* cells leave through one or two distinct regions of the prey^23^, although considerably more future work is required to prove this.

The treatment-outcomes (Fig. 7) of these prey ghosts give an insight into the elastic nature of the Gram-negative prey cell envelope; treatment with SDS of the prey ghosts from the double mutants resulted in an expansion in all dimensions of the ghosts on average. The ghosts resulting from quadruple mutant predation became longer and thinner after SDS treatment. This suggests that proteins associated with the ghost cell wall and/or the outer membrane (or the outer membrane itself), are normally holding the sacculi in a condensed, closely packed configuration. The fact that the rod-shaped prey ghosts from predation by the quadruple mutant expanded lengthwise, but contracted slightly at the width when treated with SDS shows the importance of the cross-links in holding together the rod-shape. These prey-wall cross-links are hydrolysed by the *Bdellovibrio* predatory DacB enzymes, (Bd0816, Bd3459) to yield a rounded bdelloplast during wild type predation^1^.

Prey ghost samples were analysed by SDS-PAGE followed by MALDI-QTOF and this showed predominantly that only outer membrane porins were present, suggesting that other proteins, including those of the seemingly intact inner membrane, may be degraded or reduced by the proteases of *Bdellovibrio* (Fig. 10). Thus mutant predators denude and deplete the prey interior, taking up the resulting breakdown products to complete growth and replication, however 56% of the mutant population could do this from *outside the cell wall*. The fact that wild type *Bdellovibrio* also leave behind an inner cytoplasmic membrane vesicle demonstrated that *Bdellovibrio* consume the inner contents of the prey cell without destroying the lipids of the inner membrane. Previously, it was reported that prey cytoplasmic membrane ceased to be an effective barrier to small hydrophilic molecules within 1 hr of *Bdellovibrio* invasion^24^ and *Bdellovibrio* did not penetrate the inner membrane of the prey cell during the predatory process^25^. This reinforces the reality of a bacterial sacculus as a porous structure through which (without moving wholly across it) *Bdellovibrio* can contact the prey inner membrane and cytoplasm. It degrades and takes up prey components as a sole source of nutrients for predator growth. In our experiments the mutant predators could grow and replicate whether between inner membrane and wall or the wall and outer-membrane, yet the nutrients came from the cytoplasm of the prey cells in both cases.

The structure of one of the GlcNAc deacetylases, Bd3279, shows a two domain arrangement, unique to currently determined deacetylase enzymes, wherein the active site is abutted (and partially obscured) by an all alpha domain formed by the N-terminal region of the protein. This arrangement is restricted to the prey- invasive strains of *Bdellovibrio* (HD100, Tiberius, strain W and JSS), and is lacking from epibiotic *Bdellovibrio exovorus* (that attach to, but don’t invade prey^26^). The occlusion of the Bd3279 active site by the N-terminal domain is less severe than that observed in the HP0310 deacetylase^27^, but would require conformational change to expose the active site cleft^4^. Normal mode analysis of our structure indicates that domain-based flexation/breathing may account in part for this opening^28^. Domain opening (and potentially substrate binding) may also have an effect on the ~20 amino acid long loop (that is particular to Bd3279 and its close homologues) that abuts the active site and is disordered in our structure. An extended loop at this position of the barrel can be observed in circularly-permutated members of the CE-4 family (and thus only becomes apparent in structure:structure comparisons, obscured in sequence alignments), such as that of *E. coli* PgaB^29^ which displays polymer length-dependent substrate specificity. Domain closure appears to have affected the position of two active site motifs, given that His231 and Tyr280 are in orientations incompatible with catalysis^6^, yet we observe activity with the purified protein. Several CE-4 enzyme structures have acetate in the crystallization conditions which “locks in” an active product-bound form; our Bd3279 growth condition also has acetate present but we do not see it bound–hence, there remains the significant possibility that substrate binding is regulated by domain closure. To the best of our knowledge, ours is the first structure of a “gated” enzyme shown to have peptidoglycan deacetylase activity. It is interesting to note that (by homology) no such N-terminal gating domain exists in Bd0468; hence there is either a specific need to regulate the activity of Bd3279 or Bd0468 is regulated by other means (a transcriptional basis would be unlikely given similar expression profiles of both genes). We infer that our structure-derived gating hypothesis relates to the staged nature of prey peptidoglycan metabolism by *Bdellovibrio*; activity is required in discrete time frames. The domain-based closure we observe would be amenable to regulation by proteolysis or conformational change.

In summary, the study of peptidoglycan GlcNAc deacetylases produced by predatory bacteria to act upon prey cell walls has given an unexpected insight into the tolerances of bacterial peptidoglycan sacculi for modification without changes to overall integrity. This work also suggests that two periplasmic compartments *within* and *without* the peptidoglycan layer can support predatory growth of *Bdellovibrio,* accessing prey cytoplasmic materials through the porous peptidoglycan. Our work shows how multi-enzymic predatory processes can target bacterial components for destruction, a concept relevant to the hunt for new antibiotics. In addition we have serendipitously found a way to produce emptied Gram-negative cell ghosts that contain an inner membrane vesicle, wall and some outer membrane components. These prey ghosts may be a useful test chassis for synthetic biologists building bacteria-like cells.

## Materials and Methods

### Growth of Bacterial Strains

*Bdellovibrio bacteriovorus* strain HD100^T^ was used throughout and was maintained on Ca/HEPES buffer with *E. coli* S17-1 as prey as described previously^11^,^30^. Prey strains *E. coli* S17-1*, E. coli* 083:H1, *Acinetobacter baumannii, Proteus mirabilis* and *Pseudomonas putida* were grown in YT broth for 16 hours at 37 °C with shaking at 200 rpm. Where appropriate, Ampicillin (Apollo Scientific) was used at 50 μg ml^−1^ and IPTG (isopropyl-β-D-1-thiogalactopyranoside) used for induction of fluorescent protein expression in *E. coli* at 200 μg ml^−1^.

### Generating Markerless Deletion Mutants

Markerless deletion of the *bd0468* and *bd3279* open reading frames in *Bdellovibrio bacteriovorus* strain HD100 was achieved using a modified version of Steyert and Pineiro^31^ and described in full elsewhere^32^, with multiple deletions achieved as described previously^1^,^33^. Primers used to generate a gene deletion construct for *bd0468* were (5′-3′): Bd0468-F GCAACCCGATGAATTCATCC; Bd0468-R GCTAAAAGCTTTGATCACCTGAGCCAGTTCC; Bd0468-ΔF CTTAATTGATGAACCGCCGCTAATCTAGACCCCAAAAG; Bd0468-ΔR TCAGCTCTAGATTAGCGGCGGTTCATCAATTAAGACCTCCGGT. Primers used to generate a knock-out construct for *bd3279* were (5′-3′): Bd3279-F ATCCGGAATTCTGCAGGTTCTGAATACCTGG; Bd3279-R CTGTAGCATGCCGTTAACCTCAACAGATGGC; Bd3279-ΔF AGTAGGTGTATCAGCTTCCATAGTCTAGATTAAAGTTC; Bd3279-ΔRTCAGCTCTAGACTATGGAAGCTGATACACCTACTGAGAAACGT.

### Fluorescent tagging of deacetylases

The deacetylase genes were cloned into a vector in such a way as to fuse the genes at the C-terminus with the mCherry gene, then this fusion was subcloned into the conjugable vector pK18*mobsacB* to introduce the gene fusion into *Bdellovibrio* as described previously^34^. The primers used (5′-3′) were for *bd0468*; GTACTGGAATTCTTGATGAAACGCCCTATTTC and AGTCAGGGTACCGCGGCGGTTTAGTTTGGGGA, for *bd3279;* CGCGTTGAGCTCGTGTATATGAAGAAGCTTGT and AGTCAGGGTACCTGGAAGCTGCTGCTTTCTTT.

### RNA isolation from predatory cycle and RT-PCR analysis

Synchronous predatory infections of *Bdellovibrio bacteriovorus* HD100 on *E. coli* S17-1 as well as S17-1 alone were set up as previously described^30^ with samples throughout the timecourse being taken and total RNA isolated from them. RNA was isolated from the samples using a Promega SV total RNA isolation kit with the RNA quality being verified by an Agilent Bioanalyser using the RNA Nano kit. RT-PCR was performed with the Qiagen One-step RT-PCR kit with the following reaction conditions: One cycle 50 °C for 30 mins, 95 °C for 15 mins, then 25 cycles of 94 °C for 1 min, 48 °C for 1 min, 72 °C for 2 mins, and finally a 10 mins extension at 72 °C after the 30 cycles, and finally a 4 °C hold. All experiments were carried out with at least 2 biological repeats. Primers to anneal to *bd0468* were (5′-3′): Bd0468 RT-F CAAAGGTAACGAAGCGATCC and Bd0468 RT-R AGTTCGTGGAAGTTCGGATG Primers to anneal to *bd3279* were (5′-3′): Bd3279 RT-F ACGCGTGCTTGGGAACCAGC and Bd3279 RT-R ACTTCCGCAGCCGCTTCATCG.

### Fluorescent/Time-lapse Microscopy

Epi-fluorescence microscopy was undertaken using a Nikon Eclipse E600 through a 100x objective (NA 1.25) and acquired using a Hammamatsu Orca ER Camera, the microscope was also fitted with a Prior Scientific H101A XYZ stage for revisiting the same field of view over time-lapse experiments. Images were captured using Simple PCI software (version 6.0).Time-lapse microscopy was carried out as described previously. An hcRED filter block (excitation: 550–600 nm; emission: 610–665 nm) was used for visualisation of mCherry tags. Measurement of prey exit types was from 8 biological repeats of time-lapse microscopy on pre-incubated semi-synchronous cultures. 500 μl of a 16 hours incubated predatory *Bdellovibrio* culture in Ca/HEPES buffer (see above) was concentrated 10x by centrifugation at 13,000 × *g* in a microcentrifuge for 2 minutes and mixed with 30 μl Ca/HEPES buffer and 40 μl *E. coli* backdiluted to OD_600_ of 1.0 in Ca/HEPES after being grown for 16 hours in YT broth at 37 °C with 200 rpm shaking. This resulted in a semi-synchronous infection which was pre-incubated for 3 hours at 29 °C with 200 rpm shaking, before 10 μl samples being transferred to a 1% agarose in Ca/HEPES buffer pad on a microscope slide for time lapse microscopy. Only bdelloplasts from which change in fluorescence could clearly be determined (ie fluorescence was bright enough and the focus was clear) were measured n = 112.

### Electron Microscopy

18 μl samples of prey ghosts were treated with 2 μl deionised water (control) 1%SDS (+SDS) or 1 mg ml^−1^ chicken’s egg lysozyme (Sigma) 100 mM EDTA (+ lysozyme) for 1 hour. 15 μl samples were placed on Veco copper grids, 200 mesh for 2–5 minutes, washed with PBS for 1–2 minutes and stained with 15 μl 2% phosphotungstic acid (PTA) solution pH 7.0 for 30 s to 2 minutes. Cells were observed with a JEOL 1200Ex electron microscope at 80 kV.

### SDS-PAGE analysis

30 μl prey ghost samples were added to 10 μl 4x Tris loading buffer and incubated at 105 °C for 5 minutes^35^ before being loaded onto a 12% polyacrylamide SDS gel and ran at 100 V until the sample dye reached the end of the gel. The gels were stained with Coomassie Brilliant Blue Stain and destained with 30% methanol/10% acetic acid solution. Bands were cut out of the gel and sent for LC-MS/MS analysis.

### Cloning of Bd3279 and Bd0468

Nucleotide primer pairs 5′-gtttaactttaagaaggagatatacatatgagccagatcggcagcagtgttcg-3′ and 5′-gctgcactaccgcgtggcacaagctttggaagctgctgctttcttttaacc -3′ were used to amplify the region encoding the secreted form of Bd3279 (starting at Ser24 with mutation of Gly23 to become the new N-terminal methionine, and placing a thrombin cleavable His_8_ tag on the C-terminal end of the protein; left uncleaved). The product was utilized in a second round of PCR, inserted into a modified version of the expression plasmid pET41 (Novagen, altered to remove GST) in a restriction-free process^36^. Similarly, primers 5′-gcagcggcctggtgccgcgcggcagccatatggctctgccacaggctccttccaacg-3′ and 5′-ctcagtggtggtggtggtggtgctcgagttagcggcggtttagtttggggaag-3′ were used to clone the secreted form of Bd0468 (starting at Ala26) into pET28a to give an N-terminal cleavable His_6_ tag (left uncleaved). Constructs were confirmed by sequencing, and introduced into the *E. coli* expression strain BL21 λDE3.

### Protein Expression and Purification

Cells were grown at 37 °C until reaching an OD_600_ of ~0.6, then gene expression induced with 0.2 mM IPTG for 20 hours at 20 °C. Harvested cells (approx. 14 g from 1 L cell culture in TB media for Bd3279, 8.1 g for Bd0468) were resuspended by tumbling in 45 mL resuspension buffer (20 mM imidazole pH 7.2, 0.3 M NaCl, 5% w/v glycerol and 0.1% w/v Tween20) and lysed using sonication. Unbroken cells were pelleted by centrifugation at 6000 g for 20 minutes, the supernatant clarified by a second centrifugation at 200,000 × *g* for 1 hour, and the final supernatant applied to a 1 mL Hi-Trap His column, pre-equilibrated in buffer A (20 mM HEPES pH 7.2, 0.5 M NaCl, 5% w/v glycerol and 20 mM imidazole). Fractions were eluted in a stepwise manner, using buffer A containing 40 and 300 mM imidazole,respectively. Approximately pure fractions of target protein were dialyzed overnight in buffer B (10 mM Tris pH 8.0, 100 mM NaCl, 5% w/v glycerol) and concentrated to a protein concentration of ~25 mg/mL (Bd3279) or 12.5 mg/ml (Bd0468).

### Enzyme assay of purified Bd0468 and Bd3279

Bd0468 and Bd3279 proteins were incubated with purified PG from *E. coli* D456 for 4 h at 37 °C in buffer (Bd0468: 10 mM Tris pH 7.5, 100 mM NaCl, 3.3% glycerol; Bd3279: 20 mM Tris-HCl pH 8.0). Control samples contained either no protein or no peptidoglycan. Muropeptides from the treated PG were isolated by digestion with muramidase Cellosyl as described previously^37^,^38^, and were analysed by HPLC. The structures of the muropeptide peaks of interest were assigned using linear quadrupole ion trap with combined Fourier transform ion cyclotron resonance mass spectrometry (LTQ-FT MS)^39^.

### Database deposition

Atomic co-ordinates and structure factors have been deposited in the Protein Data Bank with accession code 5JP6.

### Isolation and purification of prey ghosts

One litre predatory cultures of *B. bacteriovorus Δbd0468Δbd3279* were grown (inoculation of 50 ml overnight predatory culture into 1 L Ca/HEPES buffer with 60 ml prey culture grown 16 hours in YT broth) for 24 hours and checked by phase contrast microscopy for elimination of prey cells and proliferation of *Bdellovibrio* cells (and presence of prey ghosts). Cells and prey debris were harvested by centrifugation at 15,000 × *g* for 25 min. The pellet was resuspended in an aqueous solution of 49.4% Percoll in 0.3 M NaCl and centrifuged to form a Percoll gradient at 29,376 × *g* for 17 min; this pelleted cells and formed a band of prey ghosts in the top 1/4 top of the tube. The band was removed (2–4 ml) and added to more Percoll solution (41.4–49.4% Percoll in 0.3 M NaCl) and the procedure was repeated 4–6 times to produce a concentrated prey ghost preparation with cell contamination removed. 4 ml of this was diluted with 10 ml deionised water, centrifuged at 3,400 × *g* for 15 min. The pellet of prey ghosts was then resuspended in 1 ml deionised water at 4 °C.

### Measuring the number of residual prey ghosts per *E. coli*

To correlate the number of residual prey ghost structures seen after the *B. bacteriovorus* HD100 ∆*bd0468*∆*bd3279* strain had completed predation with the number of input *E. coli* prey, an experiment was designed to calculate the ratio of bdelloplasts (rounded prey cells infected with *Bdellovibrio*) to polystyrene beads at 30 min post-infection and compare this to the ratio of prey ghosts to polystyrene beads after the predatory cycle was completed (5 h). A semi-synchronous predatory infection (>3:1 predator:prey MOI) was set up as described previously^40^. At the 30 min and 5 hour timepoints, 38 μl was mixed with 2 μl polystyrene beads and observed by phase contrast microscopy (Nikon Eclipse E600 100x objective (NA 1.25)). Images were acquired (Hammamatsu Orca ER Camera) and analysed for number of phase bright beads compared to (at 30 min) solid phase dark bdelloplasts or (at 5 hours) more transparent phase dark prey ghost structures. The polystyrene beads were prepared as follows: 50 μl of polystyrene beads (in SDS solution) with a constant diameter of 2 μm (Microbeads, www.micro-beads.com, 2.52 × 10^9^ beads ml^−1^) was centrifuged at 17000 × *g* for 2 min. The supernatant was removed and the pellet was resuspended in 50 μl Ca/HEPES buffer. This washing process was repeated 5 times to remove all SDS. At least 3 biological repeats were carried out.

#### TMA-DPH membrane stain

A semi-synchronous predatory infection (>3:1 MOI) was set up as described previously^40^. 100 μl samples were taken at 30 min and then hourly from 2–6 hours, spun at 16,200 × *g* for 2 min, resuspended in 10 μM TMA-DPH (1-(4-Trimethylammonium-phenyl)-6-Phenyl-1,3,5-Hexatriene *p*-Toluenesulfonate)in phosphate buffered saline (PBS) and incubated in the dark for 2 min. The sample was spun at 16,200 × *g* for 2 min, the pellet washed by resuspending in 100 μl PBS, spun again at 16,200 × *g* for 2 min and finally the pellet was resuspended in 20 μl PBS, with 6 μl applied to 1% agarose in Ca/HEPES buffer on a slide for subsequent epi-flourescence microscopy as above, but with a DAPI filter block (excitation: 340–380 nm; emission: 435–485 nm).

### Crystallization and Structure Determination of Bd3279

Crystals were grown by the hanging drop method at 18 °C, using 1 microlitre of protein solution mixed with an equal volume of reservoir solution. Initial crystallization conditions were identified in 0.1 M Na cacodylate pH 6.5, 0.2 M Mg acetate, 20% w/v PEG 8000. Cryoprotection was attained by sequential addition of increments of mother liquor supplemented with 15% (v/v) ethylene glycol, followed by subsequent flash cooling in liquid nitrogen. Diffraction data were collected at beamline I02 of the Diamond Light Source, Oxford. Data were processed using XDS^41^ and SCALA, and data file manipulations performed using the CCP4 suite of programs^42^. Molecular replacement attempts utilizing PHASER^43^ with a polyalanine version of the catalytic domain of *Streptococcus pneumoniae* PgdA (residues 268–461, PDB code 2C1G^6^, yielded a clear solution, identifying one copy of Bd3279 in the asymmetric unit. The automated rebuilding procedure in PHENIX^44^ in conjunction with the relatively high resolution of the data and large solvent content (68%) resulted in the successful tracing of most of the sequence, including the N-terminal all alpha domain which is not shared with PgdA. The remaining parts of the molecule were built manually using COOT^45^ and model refinement used PHENIX^44^ and the PDB-REDO server^46^. Structural data is reported at Supporting online material Table S4.

## Additional Information

**How to cite this article**: Lambert, C. *et al*. Interrupting peptidoglycan deacetylation during *Bdellovibrio* predator-prey interaction prevents ultimate destruction of prey wall, liberating bacterial-ghosts. *Sci. Rep.* **6**, 26010; doi: 10.1038/srep26010 (2016).

## Supplementary Material

Supplementary Information

## Figures and Tables

**Table 1:** Protein bands from Fig. 10 that were analysed by mass spectrometry.

**Lane**	***Bdellovibrio*** **mutant**	**Prey**	**Top Mascot hit**	**Predicted size**
1	n/a	n/a	n/a	n/a
2	Quadruple	*E. coli* 083:H1	OMPC	41 kDa
3	Double deacetylase	*E. coli* S17-1	OMPC	40 kDa
4	Quadruple	*Proteus mirabilis*	OMPF	41 kDa
5	Quadruple	*E. coli* S17-1	OMPF	39 kDa
6	n/a	n/a	n/a	n/a
7	n/a	n/a	n/a	n/a
8	Double deacetylase	*Pseudomonas putida*	A- hypothetical lipoprotein PflA506_0906	67 kDa
			B- OprD	51 kDa
			C- OprE3	46 kDa
9	Quadruple	*Pseudomonas putida*	A- hypothetical lipoprotein PflA506_0906	67 kDa
			B- OprD	51 kDa
			C- OprE3	46 kDa
10	Double deacetylase	*Acinetobacter baumannii*	A- ompA/omp38	38 kDa
			B- ompA/omp38	38 kDa
11	Double	*Acinetobacter*	A- ompA/omp38	38 kDa
	deacetylase	*baumannii*	B- ompF	38 kDa

The top protein hits predicted by Mascot for each band are included. Predicted size is based on the whole amino acid sequence. Double deacetylase = ∆Bd0468Bd3279 mutant strain, Quadruple = ∆Bd0468∆Bd3279∆Bd0816∆Bd3459 mutant strain.

**Table 2:** Summary of the peaks shown in Fig. 12 and the reduced muropeptides in HPLC fractions obtained from *E. coli* D456 peptidoglycan treated with either Bd0468 or Bd3279 detected by LTQ-FT MS^1^.

Peak Number	Proposed Structure(s)^1^	Theoretical neutral mass (Da)	Measured neutral mass (Da)
**1**	Tetra	941.4077	941.4071
**2**	TetraTetra	1864.8049	1864.8018
**3**	TetraTetra[deAc]^‡^	1822.7943	1822.7932
**4**	TetraTetra[deAc]^‡^	1822.7943	1822.7902
	+ TetraTetra[2x deAc]	1780.7837	1780.7846
**5**	TetraTetra[deAc]^‡^	1822.7943	1822.7944
**6**	TetraTetra[deAc]^‡^	1822.7943	1822.7914

Names of muropeptides are according to Glauner^21^ Modifications: [deAc], deacetylation of GlcNAc; ^‡^ position of the deacetylated GlcNAc residue is not known.

## References

[b1] Lerner T. R. . Specialized peptidoglycan hydrolases sculpt the intra-bacterial niche of predatory *Bdellovibrio* and increase population fitness. Plos pathogens 8, e1002524, doi: 10.1371/journal.ppat.1002524 (2012). 22346754 PMC3276566

[b2] Lambert C. . Ankyrin-mediated self-protection during cell invasion by the bacterial predator *Bdellovibrio bacteriovorus*. Nature communications 6, 8884, doi: 10.1038/ncomms9884 (2015). PMC4686830 26626559

[b3] Thomashow M. F. & Rittenberg S. C. Intraperiplasmic growth of *Bdellovibrio bacteriovorus* 109J: N-deacetylation of *Escherichia coli* peptidoglycan amino sugars. J Bacteriol 135, 1008–1014 (1978). 357410 10.1128/jb.135.3.1008-1014.1978 PMC222477

[b4] Thomashow M. F. & Rittenberg S. C. Intraperiplasmic growth of *Bdellovibrio bacteriovorus* 109J: solubilization of *Escherichia coli* peptidoglycan. J Bacteriol 135, 998–1007 (1978). 357428 10.1128/jb.135.3.998-1007.1978 PMC222476

[b5] Vollmer W. & Tomasz A. The pgdA gene encodes for a peptidoglycan N-acetylglucosamine deacetylase in *Streptococcus pneumoniae*. J Biol Chem 275, 20496–20501, doi: 10.1074/jbc.M910189199 (2000). 10781617

[b6] Blair D. E., Schuttelkopf A. W., MacRae J. I. & van Aalten D. M. Structure and metal-dependent mechanism of peptidoglycan deacetylase, a streptococcal virulence factor. Proc Natl Acad Sci USA 102, 15429–15434, doi: 10.1073/pnas.0504339102 (2005). 16221761 PMC1252587

[b7] Koval S. F., Williams H. N. & Stine O. C. Reclassification of *Bacteriovorax marinus* as *Halobacteriovorax marinus* gen. nov., comb. nov. and *Bacteriovorax litoralis* as *Halobacteriovorax litoralis* comb. nov.; description of Halobacteriovoraceae fam. nov. in the class Deltaproteobacteria. Int J Syst Evol Microbiol 65, 593–597, doi: 10.1099/ijs.0.070201-0 (2015). 25406234 PMC4811658

[b8] Dori-Bachash M., Dassa B., Pietrokovski S. & Jurkevitch E. Proteome-based comparative analyses of growth stages reveal new cell cycle-dependent functions in the predatory bacterium *Bdellovibrio bacteriovorus*. Appl Environ Microbiol 74, 7152–7162 (2008). 18836011 10.1128/AEM.01736-08 PMC2592910

[b9] Rittenberg S. C. & Shilo M. Early host damage in the infection cycle of *Bdellovibrio bacteriovorus*. J Bacteriol 102, 149–160 (1970). 4908670 10.1128/jb.102.1.149-160.1970 PMC284981

[b10] Lambert C. & Sockett R. Nucleases in *Bdellovibrio bacteriovorus* contribute towards efficient self-biofilm formation and eradication of pre-formed prey biofilms. FEMS Microbiology Letters, doi: 10.1111/1574-6968.12075 (2013). PMC3593177 23297829

[b11] Morehouse K. A., Hobley L., Capeness M. & Sockett R. E. Three motAB Stator Gene Products in *Bdellovibrio bacteriovorus* Contribute to Motility of a Single Flagellum during Predatory and Prey-Independent Growth. J Bacteriol 193, 932–943, doi: 10.1128/JB.00941-10 (2011). 21148728 PMC3028683

[b12] Rotem O. . Cell-cycle progress in obligate predatory bacteria is dependent upon sequential sensing of prey recognition and prey quality cues. Proc Natl Acad Sci USA 112, E6028–6037, doi: 10.1073/pnas.1515749112 (2015). 26487679 PMC4640792

[b13] Unverdorben P. . Deep classification of a large cryo-EM dataset defines the conformational landscape of the 26S proteasome. Proc Natl Acad Sci USA 111, 5544–5549, doi: 10.1073/pnas.1403409111 (2014). 24706844 PMC3992697

[b14] Smith C. L. . Structural basis of *Streptococcus pyogenes* immunity to its NAD+ glycohydrolase toxin. Structure 19, 192–202, doi: 10.1016/j.str.2010.12.013 (2011). 21300288 PMC3056158

[b15] Holm L. & Sander C. Dali: a network tool for protein structure comparison. Trends Biochem Sci 20, 478–480 (1995). 8578593 10.1016/s0968-0004(00)89105-7

[b16] Lombard V., Golaconda Ramulu H., Drula E., Coutinho P. M. & Henrissat B. The carbohydrate-active enzymes database (CAZy) in 2013. Nucleic Acids Res 42, D490–495, doi: 10.1093/nar/gkt1178 (2014). 24270786 PMC3965031

[b17] Fadouloglou V. E. . Structure determination through homology modelling and torsion-angle simulated annealing: application to a polysaccharide deacetylase from *Bacillus cereus*. Acta crystallographica. Section D, Biological crystallography 69, 276–283, doi: 10.1107/S0907444912045829 (2013). 23385463

[b18] Arnaouteli S. . Two putative polysaccharide deacetylases are required for osmotic stability and cell shape maintenance in *Bacillus anthracis*. J Biol Chem, doi: 10.1074/jbc.M115.640029 (2015). PMC4505593 25825488

[b19] Caspi Y. & Dekker C. Divided we stand: splitting synthetic cells for their proliferation. Systems and synthetic biology 8, 249–269, doi: 10.1007/s11693-014-9145-7 (2014). 25136387 PMC4127174

[b20] Errington J. L-form bacteria, cell walls and the origins of life. Open biology 3, 120143, doi: 10.1098/rsob.120143 (2013). 23303308 PMC3603455

[b21] Glauner B. Separation and quantification of muropeptides with high-performance liquid chromatography. Analytical biochemistry 172, 451–464 (1988). 3056100 10.1016/0003-2697(88)90468-x

[b22] Tudor J. J., McCann M. P. & Acrich I. A. A new model for the penetration of prey cells by bdellovibrios. J Bacteriol 172, 2421–2426 (1990). 2185219 10.1128/jb.172.5.2421-2426.1990 PMC208878

[b23] Fenton A. K., Kanna M., Woods R. D., Aizawa S. I. & Sockett R. E. Shadowing the Actions of a Predator: Backlit Fluorescent Microscopy Reveals Synchronous Nonbinary Septation of Predatory *Bdellovibrio* inside Prey and Exit through Discrete Bdelloplast Pores. J Bacteriol 192, 6329–6335, doi: 10.1128/JB.00914-10 (2010). 20935099 PMC3008530

[b24] Cover W. H., Martinez R. J. & Rittenberg S. C. Permeability of the boundary layers of *Bdellovibrio bacteriovorus* 109J and its bdelloplasts to small hydrophilic molecules. J Bacteriol 157, 385–390 (1984). 6363383 10.1128/jb.157.2.385-390.1984 PMC215259

[b25] Burnham J. C., Hashimoto T. & Conti S. F. Electron microscopic observations on the penetration of *Bdellovibrio bacteriovorus* into gram-negative bacterial hosts. J Bacteriol 96, 1366–1381 (1968). 4879563 10.1128/jb.96.4.1366-1381.1968 PMC252461

[b26] Koval S. F. . *Bdellovibrio exovorus* sp. nov., a novel predator of *Caulobacter crescentus*. Int J Syst Evol Microbiol 63, 146–151, doi: 10.1099/ijs.0.039701-0 (2013). 22368169

[b27] Shaik M. M., Cendron L., Percudani R. & Zanotti G. The structure of *Helicobacter pylori* HP0310 reveals an atypical peptidoglycan deacetylase. Plos one 6, e19207, doi: 10.1371/journal.pone.0019207 (2011). 21559431 PMC3084791

[b28] Suhre K. & Sanejouand Y. H. ElNemo: a normal mode web server for protein movement analysis and the generation of templates for molecular replacement. Nucleic Acids Res 32, W610–614, doi: 10.1093/nar/gkh368 (2004). 15215461 PMC441506

[b29] Little D. J. . The structure- and metal-dependent activity of *Escherichia coli* PgaB provides insight into the partial de-N-acetylation of poly-beta-1,6-N-acetyl-D-glucosamine. J Biol Chem 287, 31126–31137, doi: 10.1074/jbc.M112.390005 (2012). 22810235 PMC3438944

[b30] Lambert C. . Characterizing the flagellar filament and the role of motility in bacterial prey-penetration by *Bdellovibrio bacteriovorus*. Molecular Microbiology 60, 274–286, doi: 10.1111/j.1365-2958.2006.05081.x (2006). 16573680 PMC1453311

[b31] Steyert S. R. & Pineiro S. A. Development of a novel genetic system to create markerless deletion mutants of *Bdellovibrio bacteriovorus*. Appl Environ Microbiol 73, 4717–4724 (2007). 17557848 10.1128/AEM.00640-07 PMC1951038

[b32] Capeness M. J. . Activity of *Bdellovibrio hit* locus proteins, Bd0108 and Bd0109, links Type IVa pilus extrusion/retraction status to prey-independent growth signalling. Plos one 8, e79759, doi: 10.1371/journal.pone.0079759 (2013). 24224002 PMC3818213

[b33] Lambert C. & Sockett R. E. Nucleases in Bdellovibrio bacteriovorus contribute towards efficient self-biofilm formation and eradication of preformed prey biofilms. FEMS Microbiol Lett 340, 109–116, doi: 10.1111/1574-6968.12075 (2013). 23297829 PMC3593177

[b34] Fenton A. K., Lambert C., Wagstaff P. C. & Sockett R. E. Manipulating each MreB of *Bdellovibrio bacteriovorus* gives diverse morphological and predatory phenotypes. J Bacteriol 192, 1299–1311, doi: 10.1128/JB.01157-09 (2010). 20023029 PMC2820843

[b35] Schagger H. Tricine-SDS-PAGE. Nature protocols 1, 16–22, doi: 10.1038/nprot.2006.4 (2006). 17406207

[b36] van den Ent F. & Lowe J. RF cloning: a restriction-free method for inserting target genes into plasmids. Journal of biochemical and biophysical methods 67, 67–74, doi: 10.1016/j.jbbm.2005.12.008 (2006). 16480772

[b37] Glauner B., Holtje J. V. & Schwarz U. The composition of the murein of *Escherichia coli*. J Biol Chem 263, 10088–10095 (1988). 3292521

[b38] Srikannathasan V. . Structural basis for type VI secreted peptidoglycan DL-endopeptidase function, specificity and neutralization in *Serratia marcescens*. Acta crystallographica. Section D, Biological crystallography 69, 2468–2482, doi: 10.1107/S0907444913022725 (2013). 24311588 PMC3852654

[b39] Bui N. K. . The peptidoglycan sacculus of *Myxococcus xanthus* has unusual structural features and is degraded during glycerol-induced myxospore development. J Bacteriol 191, 494–505, doi: 10.1128/JB.00608-08 (2009). 18996994 PMC2620817

[b40] Lambert C., Chang C. Y., Capeness M. J. & Sockett R. E. The first bite-maprofiling the predatosome in the bacterial pathogen *Bdellovibrio*. Plos one 5, e8599, doi: 10.1371/journal.pone.0008599 (2010). 20062540 PMC2797640

[b41] Kabsch W. Xds. Acta crystallographica. Section D, Biological crystallography 66, 125–132, doi: 10.1107/S0907444909047337 (2010). 20124692 PMC2815665

[b42] Winn M. D. . Overview of the CCP4 suite and current developments. Acta crystallographica. Section D, Biological crystallography 67, 235–242, doi: 10.1107/S0907444910045749 (2011). 21460441 PMC3069738

[b43] McCoy A. J. . Phaser crystallographic software. Journal of applied crystallography 40, 658–674, doi: 10.1107/S0021889807021206 (2007). 19461840 PMC2483472

[b44] Zwart P. H. . Automated structure solution with the PHENIX suite. Methods in molecular biology 426, 419–435, doi: 10.1007/978-1-60327-058-8_28 (2008). 18542881

[b45] Emsley P. & Cowtan K. Coot: model-building tools for molecular graphics. Acta crystallographica. Section D, Biological crystallography 60, 2126–2132, doi: 10.1107/S0907444904019158 (2004). 15572765

[b46] Joosten R. P., Joosten K., Cohen S. X., Vriend G. & Perrakis A. Automatic rebuilding and optimization of crystallographic structures in the Protein Data Bank. Bioinformatics 27, 3392–3398, doi: 10.1093/bioinformatics/btr590 (2011). 22034521 PMC3232375

[b47] Robert X. & Gouet P. Deciphering key features in protein structures with the new ENDscript server. Nucleic Acids Res 42, W320–324, doi: 10.1093/nar/gku316 (2014). 24753421 PMC4086106

