# Supplementary material for: Interrupting peptidoglycan deacetylation during Bdellovibrio predator-prey interaction prevents ultimate destruction of prey wall, liberating bacterial-ghosts
Source: Sci Rep. 2016 May 23;6:26010. doi: 10.1038/srep26010 (PMC4876506; doi:10.1038/srep26010)
Supplement: Supplementary Information [file srep26010-s1.docx]

**Supplementary Information for:**

**Interrupting peptidoglycan deacetylation during *Bdellovibrio* predator-prey interaction prevents ultimate destruction of prey wall, liberating bacterial-ghosts**

**Carey Lambert ^1,a^, Thomas R Lerner ^1,b^, Nhat Khai Bui^c,d^, Hannah Somers^a^, Shin-Ichi Aizawa^e^, Susan Liddell^f^, Ana Clark**^a,g^, **Waldemar Vollmer^c^, Andrew L Lovering^*,h^ R Elizabeth Sockett^*,a^**

^1^Contributed equally to this work. ^*^corresponding authors [liz.sockett@nottingham.ac.uk](mailto:liz.sockett@nottingham.ac.uk) +(44) 115 8230325; [a.lovering@bham.ac.uk](mailto:a.lovering@bham.ac.uk) +44 (0)121 41 45419

Author affiliations:

^a^Centre for Genetics and Genomics, School of Life Sciences, University of Nottingham, Medical School, Queen’s Medical Centre, Nottingham, NG7 2UH, UK.

^b^ The Francis Crick Institute, Mill Hill Laboratory, The Ridgeway, Mill Hill, London, NW7 1AA.

^c^The Centre for Bacterial Cell Biology, Baddiley Clark Building, Medical School, Newcastle University, Richardson Road, Newcastle upon Tyne, NE2 4AX, UK

^d^present address: Helmholtz Centre for Infection Research, Helmholtz Institute for Pharmaceutical Research, Saarland University, 66123 Saarbrücken, Germany

^e^Department of Life Sciences, Prefectural University of Hiroshima, Shobara, Hiroshima, 727-0023, Japan

^f^School of Biosciences, University of Nottingham, Sutton Bonington, Leicestershire,
LE12 5RD, UK

A-BLAST results using the Bd0468 amino acid sequence against the database of Bdellovibrionaceae and Halobacteriovoraxaceae

| Strain | Expect | Identities | Positives | Gaps | Locus tag |
| --- | --- | --- | --- | --- | --- |
| *B. bacteriovorus* HD100 | 0.0 | 290/290(100%) | 290/290(100%) | 0/290(0%) | Bd0468 |
| *B. bacteriovorus* 109J | 0.0 | 289/290(99%) | 289/290(99%) | 0/290(0%) | EP01_14245 |
| *B. bacteriovorus* Tiberius | 0.0 | 266/291(91%) | 275/291(94%) | 1/291(0%) | Bdt_0458 |
| *B. bacteriovorus* RO | 4e-67 | 116/297(39%) | 171/297(57%) | 15/297(5%) | AZI86_09770 |
| *B. bacteriovorus* RO | 5e-31 | 80/242(33%) | 117/242(48%) | 47/242(19%) | AZI86_12890 |
| *B. bacteriovorus* W | 8e-26 | 74/224(33%) | 106/224(47%) | 47/224(20%) | BDW_06420 |
| *B. exovorus* JSS | 4e-23 | 64/203(32%) | 98/203(48%) | \| 21/203(10%) \| \| --- \| | A11Q_1865 |
| *B. bacteriovorus* W | 4e-23 | 75/236(32%) | 115/236(48%) | 38/236(16%) | BDW_12005 |
| *B. bacteriovorus* EC13 | 3e-22 | 60/208(29%) | 96/208(46%) | 16/208(7%) | AZI87_04970 |
| *B. bacteriovorus* BER2 | 3e-22 | 60/204(29%) | 96/204(47%) | 16/204(7%) | AZI85_00795 |
| *B. bacteriovorus* ArHS | 4e-22 | 61/211(29%) | 98/211(46%) | 18/211(8%) | OM95_07815 |
| *B. bacteriovorus* RO | 2e-21 | 62/226(27%) | 103/226(45%) | 32/226(14%) | AZI86_03325 |
| *B. bacteriovorus* Tiberius | 4e-20 | 66/232(28%) | 102/232(43%) | 31/232(13%) | Bdt_3198 |
| *B. bacteriovorus* HD100 | 7e-20 | 64/232(28%) | 102/232(43%) | 31/232(13%) | Bd3279 |
| *B. bacteriovorus* 109J | 8e-20 | 64/232(28%) | 102/232(43%) | 31/232(13%) | EP01_01630 |
| *B. bacteriovorus* EC13 | 7e-17 | 60/226(27%) | 92/226(40%) | 36/226(15%) | AZI87_03860 |
| *B. bacteriovorus* ArHS | 9e-17 | 58/211(27%) | 87/211(41%) | 30/211(14%) | OM95_04855 |
| *B. bacteriovorus* BER2 | 1e-16 | 59/223(26%) | 89/223(39%) | 30/223(13%) | AZI85_08440 |
| *Halobacteriovorax marinus* SJ | 7e-21 | 84/324(26%) | 134/324(41%) | 68/324(20%) | BMS_1010 |

B- BLAST results using the Bd3279 amino acid sequence against the database of Bdellovibrionaceae and Halobacteriovoraxaceae

| Strain | Expect | Identities | Positives | Gaps | Locus tag |
| --- | --- | --- | --- | --- | --- |
| *B. bacteriovorus* HD100 | 0.0 | 382/383(99%) | 383/383(100%) | 0/383(0%) | Bd3279 |
| *B. bacteriovorus* 109J | 0.0 | 379/381(99%) | 380/381(99%) | 0/381(0%) | EP01_01630 |
| *B. bacteriovorus* Tiberius | 0.0 | 369/381(97%) | 377/381(98%) | 0/381(0%) | Bdt_3198 |
| *B. bacteriovorus* W | 1e-174 | 217/377(58%) | 302/377(80%) | 1/377(0%) | BDW_12005 |
| *B. bacteriovorus* ArHS | 3e-169 | 225/377(60%) | 282/377(74%) | 2/377(0%) | OM95_04855 |
| *B. bacteriovorus* BER2 | 3e-164 | 217/377(58%) | 283/377(75%) | 3/377(0%) | AZI85_08440 |
| *B. bacteriovorus* EC13 | 5e-164 | 219/377(58%) | 281/377(74%) | 3/377(0%) | AZI87_03860 |
| *B. bacteriovorus* RO | 6e-141 | 193/374(52%) | 262/374(70%) | 6/374(1%) | AZI86_03325 |
| *B. exovorus* JSS | 1e-129 | 178/375(47%) | 249/375(66%) | 22/375(5%) | A11Q_1865 |
| *B. bacteriovorus* BER2 | 4e-39 | 94/310(30%) | 162/310(52%) | 25/310(8%) | AZI85_00795 |
| *B. bacteriovorus* ArHS | 3e-38 | 94/314(30%) | 166/314(52%) | 25/314(7%) | OM95_07815 |
| *B. bacteriovorus* EC13 | 3e-38 | 92/310(30%) | 161/310(51%) | 25/310(8%) | AZI87_04970 |
| *B. bacteriovorus* HD100 | 1e-19 | 64/232(28%) | 102/232(43%) | 31/232(13%) | Bd0468 |
| *B. bacteriovorus* 109J | 1e-19 | 64/232(28%) | 102/232(43%) | 31/232(13%) | EP01_14245 |
| *B. bacteriovorus* Tiberius | 7e-19 | 63/226(28%) | 97/226(42%) | 31/226(13%) | Bdt_0458 |
| *B. bacteriovorus* RO | 7e-19 | 69/240(29%) | 101/240(42%) | 54/240(22%) | AZI86_12890 |
| *B. bacteriovorus* RO | 2e-18 | 59/222(27%) | 95/222(42%) | 33/222(14%) | AZI86_09770 |
| *B. bacteriovorus* RO | 8e-17 | 61/237(26%) | 100/237(42%) | 33/237(13%) | AZI86_18240 |
| *Halobacteriovorax marinus* SJ | 1e-17 | 72/269(27%) | 110/269(40%) | 55/269(20%) | BMS_1010 |

**Supplementary Table S1-** BLAST hits from searches with amino acids with Bd0468 (A) or Bd3279 (B). All sequenced genomes of *Bdellovibrio* have a homologue of Bd0468 (highlighted in blue) and a homologue of Bd3279 (highlighted in yellow). *B. bacteriovorus* strain RO has 2 extra homologues (highlighted in red). There is no publication yet released for this strain, so it is difficult to speculate on the function of these. The related seawater predatory bacterium *Halobacteriovorax marinus* has one homologue of these sequences. E-value cutoff for these analyses was 10^-15^.


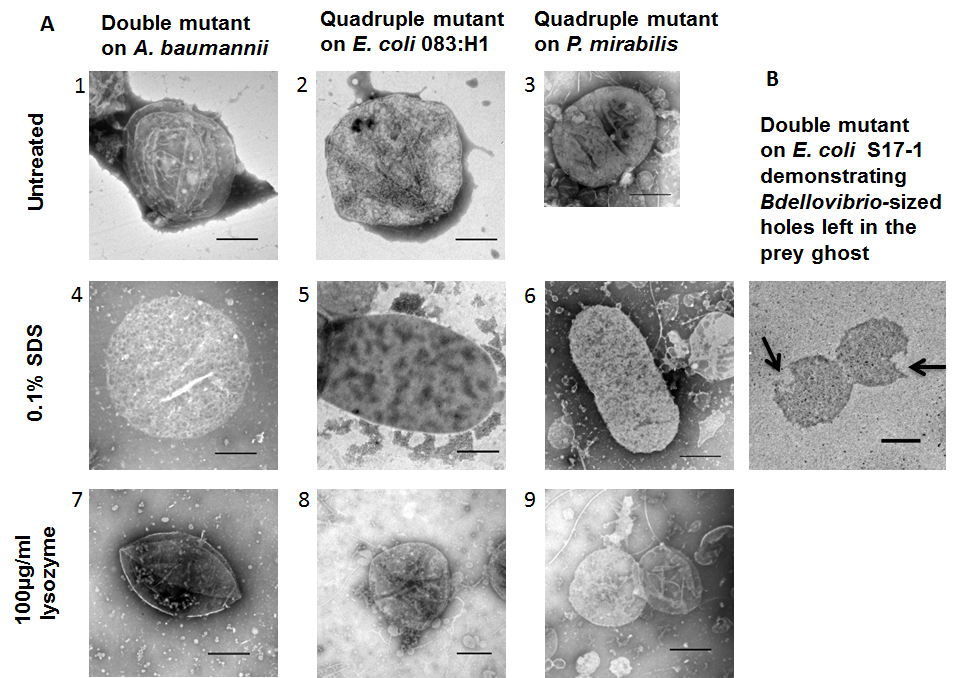


**Supplementary figure S2** Transmission electron micrographs showing ghost prey structures from different bacteria after 24 hours predation by mutant *Bdellovibrio*. Samples of ghosts were treated with final concentrations of 0.1% SDS or 100µgml^-1^ lysozyme/10mM EDTA. Double= ∆Bd0468∆Bd3279 mutant strain, Quadruple= ∆Bd0468∆Bd3279∆Bd0816∆Bd3459 mutant strain. *E = Escherichia, A= Acinetobacter, P= Proteus.* Scale bars are 500nm. **B-** Transmission electron micrograph of prey-ghosts after treatment with 0.1% SDS showing *Bdellovibrio-* sized (~0.3-0.5 µm in diameter) holes in the remains of the prey wall which may be the means by which the *Bdellovibrio* had exited the prey-ghost (arrows). Prey-ghosts were stained with 2% PTA. The scale bar represents 1 µm.


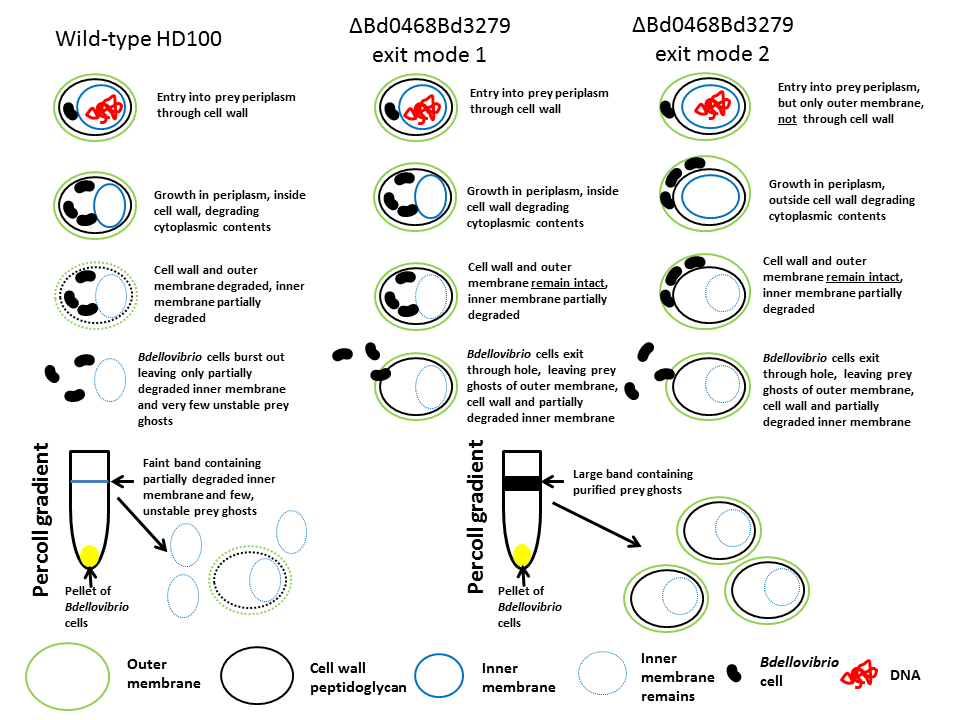


**Supplementary Figure S3** Schematic diagram showing the formation, and preparation of, purified prey ghosts by Percoll gradient centrifugation. Predation by wild-type HD100 strain resulted in very few and unstable prey ghosts and partially degraded inner membrane fragments while predation with the deacetylase double mutant (including either exit mode 1 or 2) resulted in prey ghosts consisting of outer membrane, cell wall and partially degraded inner membrane. Percoll gradients of 41.4-49.4% Percoll in 0.3 M NaCl pelleted the *Bdellovibrio* cells and gave a band higher up the gradient consisting of prey ghosts with the mutant strain, or a very faint band with the wild type strain consisting of very few and unstable prey ghosts and partially degraded inner membrane fragments.

|  | Native |
| --- | --- |
| **Data collection** |  |
| Space group | P6_1_ |
| Cell dimensions |  |
| *a*, *b*, *c* (Å) | 112.4, 112.4, 79.9 |
| α, β, γ (°) | 90, 90, 120 |
| Resolution (Å) | 1.5 (1.58-1.5)* |
| *R*_sym_ | 5.0 (-) |
| *R*_pim_ | 1.4 (35.0) |
| *I* / σ*I* | 25.1 (2.2) |
| Completeness (%) | 99.9 (99.9) |
| Redundancy | 14.0 (13.9) |
|  |  |
| **Refinement** |  |
| Resolution (Å) | 1.5 |
| *R*_work_ / *R*_free_ | 14.5/16.7 |
| R.m.s. Z-scores |  |
| rmsZ bonds | 0.66 |
| rmsZ angles | 0.79 |
| Ramachandran Plot  Total preferred (%)  Total allowed (%)  [outlier, A300, strong density]  **PDB ID code 5JP6** | 96.3%  3.4% |

*Values in parentheses are for highest-resolution shell.

**Supplementary Table S4** Data collection and refinement statistics for crystal structure of Bd3279.
